# Rootlets Hierarchical Principal Component Analysis for Revealing Nested Dependencies in Hierarchical Data

**DOI:** 10.3390/math13010072

**Published:** 2024-12-28

**Authors:** Korey P. Wylie, Jason R. Tregellas

**Affiliations:** 1 Department of Psychiatry, University of Colorado School of Medicine, Anschutz Medical Campus, Anschutz Health Sciences Building, 1890 N Revere Ct, Aurora, CO 80045, USA; 2 Research Service, Rocky Mountain Regional VA Medical Center, 1700 N Wheeling St, Aurora, CO 80045, USA

**Keywords:** eigendecomposition, multivariate statistics, hyperbolic manifold, Riemannian geometry, manifold learning

## Abstract

Hierarchical clustering analysis (HCA) is a widely used unsupervised learning method. Limitations of HCA, however, include imposing an artificial hierarchy onto non-hierarchical data and fixed two-way mergers at every level. To address this, the current work describes a novel rootlets hierarchical principal component analysis (hPCA). This method extends typical hPCA using multivariate statistics to construct adaptive multiway mergers and Riemannian geometry to visualize nested dependencies. The rootlets hPCA algorithm and its projection onto the Poincaré disk are presented as examples of this extended framework. The algorithm constructs high-dimensional mergers using a single parameter, interpreted as a p-value. It decomposes a similarity matrix from GL(m,R) using a sequence of rotations from SO(k), k≪m. Analysis shows that the rootlets algorithm limits the number of distinct eigenvalues for any merger. Nested clusters of arbitrary size but equal correlations are constructed and merged using their leading principal components. The visualization method then maps elements of SO(k) onto a low-dimensional hyperbolic manifold, the Poincaré disk. Rootlets hPCA was validated using simulated datasets with known hierarchical structure, and a neuroimaging dataset with an unknown hierarchy. Experiments demonstrate that rootlets hPCA accurately reconstructs known hierarchies and, unlike HCA, does not impose a hierarchy on data.

## 1. Introduction

Nested hierarchies occur frequently in nature and in scientific datasets. In these naturally occurring hierarchies, the number of elements merged or subdivided at each level is rarely constant. Instead, this number varies depending on the structure of the hierarchy. For example, in social networks, groups of individuals often feature cliques of varying sizes, with smaller subgroups within each. In the phylogenetic tree of life, different ancestral species have differing numbers of offspring and descendants. In neuroscience, the primary visual cortex contains a large and widely varying number of neurons and is itself a branch of the larger visual processing system.

In each of these examples, the degree of branching is a random integer. However, the most common method of analyzing hierarchically structured data, hierarchical clustering analysis (HCA), applies a fixed two-way merger or subdivision at each level [1]. HCA algorithms cannot adapt the number of variables merged based on the structure of the data, to allow for adaptive multiway mergers. Consequently, the underlying hierarchy can be poorly estimated or distorted by the limitations of HCA. Additionally, HCA always imposes a hierarchical structure on all input data and is unable to effectively parse non-hierarchical data, due to the same limitation.

The current work overcomes this limitation by extending hierarchical principal component analysis (hPCA) to allow the construction of multiway mergers. The original “treelets” hPCA algorithm [2] shares many similarities with HCA, including fixed sizes of mergers. At each level, the treelets hPCA algorithm selects a pair of variables for a two-way merger, as in HCA. The selected variables are then merged using a local PCA, creating a new variable as the leading principal component (PC). Subsequent levels of the treelets hPCA algorithm then merge this leading PC with other variables. Since the treelets hPCA algorithm closely followed the framework of HCA, it features the same strengths and weaknesses shared by other HCA algorithms, including fixed two-way mergers.

In contrast, the novel “rootlets” hPCA algorithm introduced below allows for multiway mergers. Specifically, the eigenstructure of each local PCA is used to construct criteria for multiway mergers. Multivariate statistical analysis is then used to adjust the criteria for the presence of noise in the data. The resulting hPCA algorithm thereby constructs adaptive multiway mergers. As demonstrated below, the rootlets algorithm groups variables into clusters while minimizing the number of distinct eigenvalues in each group. By utilizing the algebraic and geometric properties of PCA, properties not shared by HCA, rootlets hPCA is able to overcome the limitations of HCA.

Analyses of hierarchies from large datasets can be facilitated with suitable visualization methods. When available, such methods allow for the easy identification of network features, as well as a side-by-side comparison of results from different groups of subjects. For HCA, the standard visual display is a branching dendrogram with mergers displayed along the vertical axis in an inverted U shape [1]. This display rapidly becomes densely overplotted for large hierarchies.

To overcome this difficulty, we demonstrate how results from hPCA can be directly mapped onto a compact and low-dimensional hyperbolic space. Intuitively, the resulting display can be thought of as flattening the branches and leaves of a plant specimen between the pages of book. The result is a visually efficient and enriched method of displaying even very large hierarchies, where important features are easily distinguished without being overwhelmed with extraneous information. This mapping results from the geometric properties of hPCA, combined with the close relation between hyperbolic space and hierarchical data structures. Specifically, hyperbolic space is the natural underlying geometry for the analysis of hierarchical datasets. In fact, tree-like data structures can be viewed as a discrete approximation of an underlying smooth hyperbolic metric space. This connection will be systematically developed in the current work into a visualization algorithm used to map the output of rootlets hPCA onto the Poincaré disk.

The main contribution of this paper is to develop the novel unsupervised learning method, the rootlets hPCA algorithm, and analyze its output using spectral analysis. Additional contributions include developing a unique visualization method for hPCA and bridging the gap between the novel hPCA algorithm and existing machine learning literature, by highlighting the role hyperbolic geometry plays in both. Lastly, by demonstrating how hPCA maps data into a hyperbolic space, this analysis demonstrates how hPCA can be viewed as a manifold learning algorithm, further distinguishing it from clustering or parcellation algorithms such as HCA.

Recent work has emphasized the potential utility of hyperbolic geometry for the unsupervised learning of abstract concepts hierarchies [3] and low-dimensional stochastic embeddings of complex datasets [4–6]. Additionally, hyperbolic geometry has been applied to study communication and routing in complex networks [7,8], and to graph theoretic properties of complex networks [9]. Lastly, methods to train neural networks on hyperbolic manifolds have been developed [10].

Subsequent sections are organized as follows: Section 2.1 provides general background on PCA. Section 2.2 describes hPCA in general and introduces the rootlets hPCA algorithm. Section 2.3 describes the multivariate statistical test applied in the algorithm. Section 2.4 analyzes the rootlets algorithm using eigendecomposition. Section 2.5 develops a visualization method for hPCA output. Section 3 compares rootlets hPCA to standard HCA on simulated examples and neuroimaging datasets.

Notation: All vectors are column vectors unless otherwise noted, including vectors of random variables. Following this convention, matrices where the rows are the primary elements of interest are denoted concisely as $A={\left[a_{1}^{T},\ldots ,a_{m}^{T}\right]}^{T}$. Set-builder notation is used in the pseudo-code for Algorithms 1 and 2, with {x,y}\{y} denoting the removal of element {y} from the set. Groupings of similar variables are termed *clusters* and indexed by subscripts (e.g., $c_{a}$ and $c_{b}$ denote separate clusters of variables). GL(m,R) is the group of m×m invertible matrices, and SO(k) is the group of k×k rotation matrices. Section 2.5 assumes familiarity with concepts from Riemannian geometry and hyperbolic manifolds. Relevant concepts are developed in greater detail in the Supplementary Materials, including a construction of the contents of Section 2.5 in greater depth.

## 2. Global, Local, and Hierarchical Principal Component Analysis

### 2.1. Principal Component Analysis Background

Let the m data variables $x_{i}$ be formatted as vectors in $R^{n}$, mean-subtracted and concatenated into the data matrix $X={\left[x_{1}^{T},\ldots ,x_{m}^{T}\right]}^{T}$. PCA constructs an orthonormal basis set $\left\{v_{j}\right\}$ by diagonalizing the covariance matrix $\Sigma =XX^{T}$ as

(1) $$V^{T}\Sigma V=\left[\begin{array}{cccc} \lambda_{1} & 0 & \cdots & 0\\ 0 & \lambda_{2} & \cdots & 0\\ \vdots & \vdots & \ddots & \vdots \\ 0 & 0 & \cdots & \lambda_{k} \end{array}\right]=\Lambda ,$$

where $V=\left[v_{1},\ldots ,v_{k}\right]$ is a matrix with eigenvectors $v_{j}$ in columns, Λ is a diagonal matrix of eigenvalues $\lambda_{1}\geq \lambda_{2}\geq \ldots \geq \lambda_{k}\geq 0$, and k=min(m,n). The principal components (PCs) are then obtained by projecting the data onto the basis vectors as $U=V^{T}X$. The leading principal component PC_1_, denoted as $u_{1}$ in the matrix $U={\left[{u_{1}}^{T},\ldots ,{u_{k}}^{T}\right]}^{T}$, is the direction of maximum shared variance in the data, while PC_2_ is the largest direction of shared variance orthogonal to PC_1_. Additionally, as detailed in Appendix A, PCA constructs a coordinate system containing the input variables and output PCs. This coordinate system can be used to construct a low-dimensional visualization of PCA results, termed a *biplot*. In Section 2.4, PCA biplots will be extended to develop an algorithm used to plot hPCA results.

The above discussion implicitly used all available data variables as input and is thus a *global* PCA with respect to the entire dataset. In contrast, a *local* PCA applies to a subset of variables within the dataset. Notably, all of the above statements and calculations hold for a local PCA, provided care is taken to limit the scope of their use to the input variables and output PCs.

### 2.2. Hierarchical Principal Component Analysis Algorithms

Hierarchical PCA is a multi-resolution analysis technique, similar to wavelets analysis and HCA [2]. Hierarchical PCA represents the internal structure of data as orthonormal basis functions, structured as a hierarchical tree. Specifically, hPCA analyses structure the data at different scales using a series of sparse orthogonal transforms $V_{l}$ on the covariance matrix Σ. Each $V_{l}$ is a rotation matrix that applies a local PCA to a subset of the data variables while leaving all other variables unchanged. If V is the k-by-k rotation matrix from the local PCA for level l, then by rearranging the rows and columns so that the k variables form a block, $V_{l}$ can be represented by the sparse matrix

(2) $$V_{l}=\left[\begin{array}{ccc} I_{a} & 0 & 0\\ 0 & V & 0\\ 0 & 0 & I_{b} \end{array}\right],$$

where $I_{a},\;I_{b}$ are a-by-a and b-by-b identity matrices such that a+k+b=m and k≪a+b.

The first hPCA level merges a subset of variables, selected for their similarity to each other relative to all other variables. At level l=1, after applying the local PCA, the current state of the variables is $X_{1}=V_{1}^{T}X$ with similarity matrix $\Sigma_{1}=V_{1}^{T}\Sigma V_{1}$. The next level then selects a subset of rows of $X_{1}$ based on their similarity in $\Sigma_{1}$ and applies a local PCA, resulting in the updated similarity matrix $\Sigma_{2}=V_{2}^{T}V_{1}^{T}\Sigma V_{1}V_{2}$. At level l, the similarity matrix is of the form

(3) $$\Sigma_{l}=V_{l}^{T}\cdots V_{1}^{T}\Sigma V_{1}\cdots V_{l}.$$

The consecutive rotations $\left\{V_{1},V_{1}V_{2},V_{1}V_{2}V_{3},\ldots \right\}$ form a sequence of orthogonal basis functions with increasing non-localized support. The corresponding PCs $U_{l}=V_{l}^{T}\ldots V_{1}^{T}X$ are coordinate vectors. Furthermore, since only leading PCs from previous levels are merged in hPCA algorithms, individual $V_{l}$ overlap in only one variable. Consequently, the sequence of mergers creates a branching hierarchical tree.

Notably, unlike the hierarchy constructed by HCA, hPCA constructs a new variable at each level and a locally Euclidean coordinate system. This locally linear property, in combination with the (minimal) overlap between rotations, allows hPCA algorithms to approximate non-linear manifolds. As a concrete biological example, the bark of a tree is a highly curved surface, with leaves as node endpoints grouped by branch nodes. While HCA is able to identify groups of leaves on the same branch, it is not able to locate their shared branch node on the bark surface. In contrast, hPCA can identify groups of leaf nodes on the same branch and, additionally, locate the branch nodes on the tree bark surface by using local coordinate charts constructed by the sequence of local PCA charts.

The original *treelets* hPCA algorithm [2] summarizes relationships between variables in the data as a series of binary mergers. Each treelets hPCA level applies a two-dimensional local PCA to a pair of variables, selected using a similarity measure.

The novel *rootlets* hPCA algorithm extends hPCA to allow for adaptive multiway mergers using multivariate statistical criteria. It groups similar variables into a series of nested clusters, with each cluster size adapted to the underlying structure of the data based on the trailing eigenvalues of the local PCA.

To develop the rootlets hPCA algorithm, it is first necessary to define criteria on the eigenstructure and associated terminology. Let $\lambda_{1}\geq \lambda_{2}\geq \ldots \geq \lambda_{k}\geq 0$ be the eigenvalue sequence of a local PCA. Three definitions are applied in rootlets hPCA:

- The *cluster defining criteria* is $\lambda_{1}>\lambda_{2}=\lambda_{3}=\ldots =\lambda_{k}$;
- The *cluster branching criteria* is that $\lambda_{i}>\lambda_{j}$ for some i and j in 2≤i<j≤k;
- A *branched* cluster consists of a local PCA with static set of input variables. Branched clusters cannot be expanded with additional variables. However, the leading PC of a branched cluster can be merged with other leading PCs to form a new cluster.

The cluster defining and cluster branching criteria are disjoint and complementary on the space of positive semi-definite eigenvalue sequences with $\lambda_{1}\neq \lambda_{2}$. In rootlets hPCA, these criteria determine whether or not to expand an existing local PCA by merging additional variables. Specifically, as long as the input variables from two or more local PCAs can be combined into a single local PCA while maintaining the cluster defining criteria, the encompassed variable sets are merged and the local PCA is expanded.

Once the cluster branching criteria are triggered, the local PCAs are branched. The branching process involves three steps:

1. Individual variable sets from the input local PCAs are fixed in their pre-merged state;
2. All variables in the branched clusters are then removed from the analysis;
3. Two new variables are constructed as the leading PCs of each of the local PCAs (in their pre-branched state, fixed in step 1). These new variables then form a new cluster.

With the above definitions, the rootlets hPCA algorithm can be described in detail, summarized as the pseudo-code in Algorithm 1.

**Algorithm 1:** Rootlets Hierarchical PCA

1.	**Input:** Data vectors ${\left\{x_{i}\right\}}_{i=1,\ldots ,m}$, cluster-wise similarity measure $Sim\left(c,c^{'}\right)$,	
	k-dimensional principal component analysis ${PCA}_{k}\left(x,x^{'},\ldots \right)$,	
	statistical test $ESET\left(\lambda_{2},\lambda_{3},\ldots \right)$, and p-value cutoff parameter p_cutoff.	
2.	$𝒜\leftarrow {\left\{x_{i}\right\}}_{i=1,2,\ldots ,m}$	# initialize active clusters set with all data vectors
3.	𝒯←Ø	
4.	**For** l←1,2,…,m-1 **Do**	
5.	$c_{i},c_{j}\leftarrow arg\;max\;Sim\left(c_{a},c_{b}\right)$	# find max similarity between PC1s of clusters
	$c_{a},c_{b}\in 𝒜$	
6.	$\lambda \leftarrow {PCA}_{k}\left(c_{i}\cup c_{j}\right)$	# get eigenvalues
7.	$p\text{value}\leftarrow ESET\left(\lambda_{2},\ldots ,\lambda_{k}\right)$	# test trailing eigenvalues for equality
8.	**If** *p*value ≥ p_cutoff	
9.	$𝒜\leftarrow \left\{𝒜\backslash \left\{c_{i}\right\}\right\}\backslash \left\{c_{j}\right\}$	# remove individual clusters
10.	$c_{l}\leftarrow c_{i}\cup c_{j}$	
11.	$𝒜\leftarrow 𝒜\cup \left\{c_{l}\right\}$	
12.	**Else**	# branch clusters & merge PC1s
13.	$\left(V_{i},\lambda_{i},u_{1i}\right)\leftarrow {PCA}_{k}\left(c_{i}\right)$	# get eigendecomposition, PC1 for cluster i
14.	$𝒯\leftarrow 𝒯\cup \left\{\left(V_{i},\lambda_{i},u_{1i}\right)\right\}$	# fix cluster i
15.	$\left(V_{j},\lambda_{j},u_{1j}\right)\leftarrow {PCA}_{k}\left(c_{j}\right)$	# get eigendecomposition, PC1 for cluster j
16.	$𝒯\leftarrow 𝒯\cup \left\{\left(V_{j},\lambda_{j},u_{1j}\right)\right\}$	# fix cluster j
17.	$𝒜\leftarrow \left\{𝒜\backslash \left\{c_{i}\right\}\right\}\backslash \left\{c_{j}\right\}$	
18.	$𝒜\leftarrow 𝒜\cup \left\{u_{1i},u_{1j}\right\}$	# create new active cluster from PC1s
19.	**End If**	
20.	**End For**	
21.	𝒯←𝒯∪{(I,1,𝒜)}	# add remaining cluster of PC1s, identity rotation and scaling
22.	**Return:** tree 𝒯	

The pseudo-code in Algorithm 1 is initialized with all data variables as singleton clusters, creating the active clusters set (line 2). Subsequent levels of the algorithm will merge elements of the active clusters set, to form more extensive clusters capturing increasingly global features of the data (lines 4 to 19). Similarities between active clusters are measured as the scaled similarity between their leading PCs, as detailed in Theorem 3 below. When calculating similarities with clusters of a single variable, the leading PC of the cluster is defined as this singleton variable. Paired clusters are prioritized for merging based on this cluster-wise similarity (line 5). This procedure selects pairs of clusters with similar directions of shared variance, maximizing similarities between individual variables within and between the individual clusters considered for merging. Consequently, cluster sizes are expanded to the maximum extent while maintaining the cluster defining criteria. Statistical criteria on the eigenvalues, detailed in Section 2.3 below, are used to estimate the probability that the cluster defining criteria is true in the presence of noise (line 7). Whenever the cluster defining criteria is not satisfied for a pair of highly similar clusters, the cluster branching criteria are then invoked (line 12). The pair of clusters are branched, that is, fixed in their pre-merged state (lines 13 to 16) and removed from consideration by the algorithm (line 17). A new branched cluster is created encompassing their individual leading PCs (line 18), allowing their shared variance to contribute to subsequent mergers of the hierarchy. Since the total number of clusters decreases by one at each level, the total number of levels is the number of data variables minus one, m-1.

The branching process constructs new variables as the leading PC of leading PCs from previous local PCAs. The new variables thus feature increasingly non-localized support in the data, representing the shared variance of an increasing number of data variables. This results in a local, multiscale decomposition of the data. Relationships between the different variables and scales in the multiscale decomposition are determined by the rotation matrices $V_{l}$. This feature of rootlets hPCA will be developed into the visualization algorithm in Section 2.4.

The computational complexity of the rootlets algorithm is dependent on the sizes of the local PCA at each level. However, since only the eigenvalues and leading PC of a small subset of the total variables are calculated at each level (i.e., k≪m), the actual running time is much less than the worst-case scenario and is comparable to the treelets hPCA or HCA algorithms. The complexity of these algorithms is s+O(lm), where l is the number of levels, m is the number of variables, and s is the cost of computing the similarity measure between data variables. If simple correlations are used, the cost of computing the sample covariance matrix is $s=O\left(min\left(nm^{2},mn^{2}\right)\right)$, where n is the number of observations per variable. If the maximum possible number of levels are constructed, l=m-1. The computational complexity of the rootlets algorithm is then near $O\left(m^{2}\right)$, provided the cost of calculating the local PCAs is negligible (i.e., k≪m). To minimize the cost of calculating local PCAs, the similarity measure applied deprioritizes the formation of large clusters, as discussed in Section 2.4 below. Additionally, the branching process reduces large clusters to k=2 as the algorithm progresses. In the case where the size of the largest cluster $k_{max}$ is non-negligible, the cost of the local PCA for this level is then $O\left({k_{max}}^{3}\right)$ and the overall complexity of the rootlets algorithm is then $O\left(m^{2}+k_{max}3\right)$. For all applied datasets in Section 3, $k_{max}3\leq m^{2}$ and the computational complexity is $O\left(m^{2}\right)$.

### 2.3. The Equality of Smallest Eigenvalues Test

The rootlets algorithm applies a multivariate statistical criterion to construct adaptive multiway mergers. Specifically, the Equality of Smallest Eigenvalues Test (ESET) [11] hypothesizes that the k variables X input to the local PCA are composed of independent systemic and noise parts as X=ϒ+ε, with covariance matrices,

(4) $$\begin{array}{c} \Psi =E\left\{{ϒϒ}^{T}\right\},\\ \sigma^{2}I=E\left\{\varepsilon \varepsilon^{T}\right\},\\ \Sigma =E\left\{XX^{T}\right\}=\Psi +\sigma^{2}I, \end{array}$$

where Ψ is a positive semidefinite of rank p<k,I is the k-by-k identity matrix, and $\sigma^{2}>0$. The eigendecomposition of Σ then features an eigenvalue $\sigma^{2}$ of multiplicity k-p. The ESET null hypothesis is that the trailing k-p population eigenvalues are equal, $\lambda_{p+1}=\lambda_{p+2}=\ldots =\lambda_{k}$. The test statistic from n observations is based on the ratio of the geometric and arithmetic means, after scaling to the number of observations and eigenvalues tested. It is calculated as

(5) $$-\left(n-1\right)\log\prod_{i=p+1}^{k}\lambda_{i}+\left(n-1\right)\left(k-p\right)\log\frac{\sum_{i=p+1}^{k}\;\lambda_{i}}{k-p}.$$

The above statistic asymptotically converges to a $\chi^{2}$-distribution with ½(k-p+2)(k-p-1) degrees of freedom, allowing for a parametric statistical test and p-value. For simplicity, if k=2, the ESET null hypothesis is considered true with probability 1 since $\lambda_{k}$ is then equal to itself.

Within the rootlets algorithm, ESET with p=1 is used to estimate the probability that the cluster defining criteria is true in the presence of added i.i.d. noise. The ESET null hypothesis is the cluster defining criteria. The alternative hypothesis is the cluster branching criteria. The rootlets p-value cutoff parameter is compared to the exact p-value from the $\chi^{2}$-distribution. The null hypothesis is accepted if the exact p-value is greater than the p-value cutoff parameter, indicating any differences between the k-1 smallest eigenvalues are attributable to noise. The rejection of the null hypothesis indicates that differences between eigenvalues are systemic, triggering the cluster branching criteria.

A low p-value cutoff parameter in the rootlets algorithm will facilitate the acceptance of the ESET null hypothesis. This will result in larger clusters and branches, but fewer branches in total. A high p-value cutoff will cause the null hypothesis to be rejected more easily, resulting in smaller clusters and more frequent branching. In this sense, the p-value cutoff acts as a resolution parameter for the algorithm.

If the ESET null hypothesis is always rejected (p-value cutoff = 1), a new branch will always be created at every merger of size k>2. In this case, the rootlets algorithm creates a series of binary mergers, as in the original treelets hPCA algorithm [2]. If the null hypothesis is always accepted (p-value cutoff = 0), all input variables will be grouped into a single cluster. In this case, the rootlets algorithm then defaults to a single global PCA.

The ESET can be applied to the output of the rootlets hPCA to provide support for an absence of hierarchy, in the sense that any shared variance in the data is indistinguishable from i.i.d. noise. Specifically, if the ESET null hypothesis with rank parameter p=0 applied to the final level of the rootlets hPCA is accepted, this indicates all variables in the top-level cluster are i.i.d. If, in addition, the ESET null hypothesis was also accepted for all hPCA levels, this indicates no evidence of clustering was found in the data.

For example, if the null hypothesis with p=1 is accepted for all rootlets levels, the algorithm groups all input variables into a single cluster. If the ESET null hypothesis with p=0 is then accepted for this final cluster, any apparent correlations are attributed to random noise. In this case, no hierarchy was found in the data, with a degree of certainty determined by the rootlets p-value cutoff parameter with a suitable correction for multiple comparisons.

### 2.4. Analysis of the Rootlets hPCA Algorithm

The rootlets algorithm is defined as a series of local PCAs, with similarities calculated as correlations between leading PCs, and statistical inference on eigenvalue sequences used to construct clusters of variables. Spectral analysis methods from linear algebra can be used to gain insights into the behavior of the algorithm in terms of the types of clusters it constructs, the ordering in which clusters are constructed, and the relationship between inter-cluster similarity measures and features of the data correlation matrix.

Proposition 1 below shows that the cluster defining and branching criteria limit the complexity of the eigenvalue sequences constructed by rootlets hPCA.

#### Proposition 1.

*All clusters of size* k≥2 *constructed by the rootlets hPCA algorithm have eigenvalue sequences featuring one or two distinct values, structured as* $\lambda_{1}\geq \lambda_{2}=\ldots =\lambda_{k}$.

Proposition 1 is proved using induction in Appendix B. The proof involves considering the eigenvalue sequences of all possible mergers constructed by hPCA, including mergers with singleton clusters. As detailed in the proof, the relatively simple eigenvalue sequences of rootlets hPCA clusters are a consequence of the branching process.

Lemma 1 gives a general form of all positive semi-definite matrices constructed by hPCA.

#### Lemma 1.

*Let* Σ *be a symmetric matrix with eigenvalues* $\lambda_{1}\geq \lambda_{2}=\ldots =\lambda_{k}\geq 0$, *with repeated eigenvalue* λ. *Then the general form of* Σ *is*

(6) Σ=Ψ+λI.

*If* $\lambda_{1}>\lambda_{2},\;then\;\Psi$ *is a matrix of rank 1. If* $\lambda_{1}=\lambda_{2}=\lambda$, *then* Ψ *is matrix of zero rank. Furthermore, the eigenvectors of* Σ *are the eigenvectors of* $\Psi ,\;and\;the\;eigenvalues\;\lambda_{i}\;of\;\Sigma$ *are each equal to an eigenvalue of* Ψ *plus* λ.

The proof of Lemma 1 is given in Appendix C.

Lemma 2 is a refinement of the matrix Σ in Equation (6) above, to the case where the diagonal elements are all equal to 1.

#### Lemma 2.

*Let* Σ *be a symmetric matrix with eigenvalues* $\lambda_{1}\geq \lambda_{2}=\ldots =\lambda_{k}\geq 0\;and\;diagonal\;elements\;\Sigma_{ii}=1$. *If* $\lambda_{1}>\lambda_{2}$, *the general form of* Σ *is*

(7) $$\Sigma =vvv^{T}+\lambda I,$$

*where* v *is a k-vector with elements* $\pm k^{\left(-1/2\right)}\lambda =\lambda_{2}=\ldots =\lambda_{k}$ *is the repeated eigenvalue, and* $v=\lambda_{1}-\lambda$. *If* $\lambda_{1}=\lambda_{2},\;then\;\Sigma =I$.

The proof of Lemma 2 is given in Appendix D.

Theorem 1 then applies Lemma 2 to correlation matrices, to give an exact form for correlation submatrices constructed by hPCA. The positive semi-definite matrix product $x_{i}^{T}x_{j}=\left\langle x_{i},x_{j}\right\rangle$ is a consequence of selecting variables for merging in hPCA using a signed similarity measure, which prioritizes positive similarities for merging into clusters.

#### Theorem 1.

*Let* Σ *in* Lemma 2 *be decomposed as* $\Sigma =XX^{T}/(n-1),\;where\;X={\left[x_{1}^{T},\ldots ,x_{k}^{T}\right]}^{T}$ is a k-by-n matrix. If $\left\langle x_{i},x_{j}\right\rangle \geq 0$ for all i,j≤k, *then*

(8) $$\Sigma =r11^{T}+(1-r)I,$$

*where* 0≤r≤1 *and* 1 *is a k-vector of ones. Furthermore, if* $\lambda_{1}>\lambda_{2}$, *then* r *is the scaled difference between the leading and repeated eigenvalues of* Σ *as* $r=\left(\lambda_{1}-\lambda \right)/k$. *If* $\lambda_{1}=\lambda_{2}=\lambda$, *then* r=0.

The proof of Theorem 1 is given in Appendix E.

Theorem 2 demonstrates that the converse of Theorem 1 is true, demonstrating the close relationship between the repeated eigenvalue structure and submatrices within the data correlation matrix.

#### Theorem 2.

*Let* R *from* $R^{k\;\times \;k}$ *be structured as*

(9) $$R=r11^{T}+(1-r)I,\;0\leq r\leq 1,$$

*with eigenvalue sequence* $\lambda_{1},\ldots ,\lambda_{k}$, and *leading eigenvector* 1*. Then,* $\lambda_{1}=(k-1)r+1$*, and* $\lambda_{2}=\ldots =\lambda_{k}=1-r$. Furthermore, if r≠0 *and* r≠1*, then* $\lambda_{1}>\lambda_{2}$ *and* $v_{1}$ *is a scaled vector of ones as* $v_{1}=k^{\left(-1/2\right)}1$.

The proof of Theorem 2 is given in Appendix F.

Theorem 1 and Theorem 2 show that, if the variables within a cluster are all positively correlated, the cluster defining criteria is identical to the simple block structure,

(10) $$R=\left[\begin{array}{cccc} 1 & r & \cdots & r\\ & 1 & \cdots & r\\ & & \ddots & \vdots \\ & & & 1 \end{array}\right],0\leq r\leq 1.$$

The above blocks will be located along the diagonal of the data correlation matrix. Theorem 3 below builds upon these results, to construct a cluster-wise similarity measure related to the off-diagonal blocks in the data correlation matrix. If $R_{a},R_{b}$ are two clusters being considered for merging, then their combined correlation matrix is

(11) $$R=\left[\begin{array}{cc} R_{a} & R_{ab}\\ R_{ab}^{T} & R_{b} \end{array}\right]=\left[\begin{array}{cccccccc} 1 & r_{a} & \cdots & r_{a} & & & & \\ & 1 & \cdots & r_{a} & & & R_{ab} & \\ & & \ddots & \vdots & & & & \\ & & & 1 & & & & \\ & & & & 1 & r_{b} & \cdots & r_{b}\\ & & & & & 1 & \cdots & r_{b}\\ & & & & & & \ddots & \vdots \\ & & & & & & & 1 \end{array}\right],0\leq r_{a},r_{b}\leq 1.$$

The cluster-wise similarity measure constructed in Theorem 3 is the average of $R_{ab}$ in (11).

#### Theorem 3.

*Let* $R_{a},\;R_{b}$ *be matrices structured as in* Lemma 2 *with* a *and* b *rows, respectively. Let* $R_{a}$ *and* $R_{b}$ *have decompositions* $R_{a}=X_{a}X_{a}^{T}/(n-1)$ *and* $R_{b}=X_{b}X_{b}^{T}/(n-1)$, *and let* $R_{ab}=X_{a}X_{b}^{T}/(n-1)$, *where* $X_{c}$ *are c-by-n matrices with rows* $x_{i}^{T}$. *Let* $u_{1a},\;u_{1b}$ *be the leading principal components of* $X_{a},\;X_{b}$, *respectively, with associated eigenvalues* $\lambda_{1a}$ *and* $\lambda_{1b}$. *Then* $\left\langle u_{1a},u_{1b}\right\rangle /(n-1)$ *is equal to the sum of the elements of* $R_{ab}$ *scaled by* $(ab{)}^{1/2}$, *and* $\left\langle u_{1a},u_{1b}\right\rangle /(\left(ab{)}^{1/2}(n-1\right))$ *is equal to the average of the elements of* $R_{ab}$.

The proof of Theorem 3 is given in Appendix G.

Proposition 1 and the above theorems show that the rootlets algorithm constructs clusters corresponding to diagonal blocks of the data correlation matrix. Since the cluster-wise similarity measure in Theorem 3 is signed, initial non-singleton clusters will be two positively correlated variables (provided any positive correlations exist between data variables). Subsequent levels of this algorithm will expand these clusters by appending additional positively correlated variables. Any block of the data correlation matrix structured as in Equation (10) has submatrices as in Equation (11), with intra-cluster correlations $r_{a}=r_{b}$ and equal inter-cluster similarities. Once the rootlets algorithm constructs any part of this cluster, indicating that the intra-cluster correlation is the maximum similarity, all other intra- and inter-cluster similarities are also the maximum similarity by Theorem 3. Consequently, the subsequent levels will construct the cluster in its entirety. If a nested block structure is present in the data, cluster branching and branch merging will be used to construct a sequence of clusters from leading PCs, representing the shared variances within each nested block. Lastly, since the cluster-wise similarity is the covariance between leading PCs scaled to cluster sizes, it deprioritizes the formation of large clusters. Furthermore, this similarity is equal to the average of the off-diagonal block elements by Theorem 3 and is therefore independent of cluster size.

It is possible to generalize the above analysis to encompass clusters with negative correlations between variables. Such clusters are consistent with the general form of the cluster defining criteria as stated in Lemma 2. However, the signed similarity measure deprioritizes any negative correlations between variables in favor of clusters of positively correlated variables, as in Theorems 1 and 2. Lastly, in the case of random variables, the above theorems are asymptotically true. The denominator (n-1) in the above results from finite sample sizes drawn i.i.d. from a larger population. In the limit as n goes to infinity, all expressions in which (n-1) appears will converge to population covariances and correlations.

### 2.5. Visualization of Hierarchical PCA

**Figure 1.**
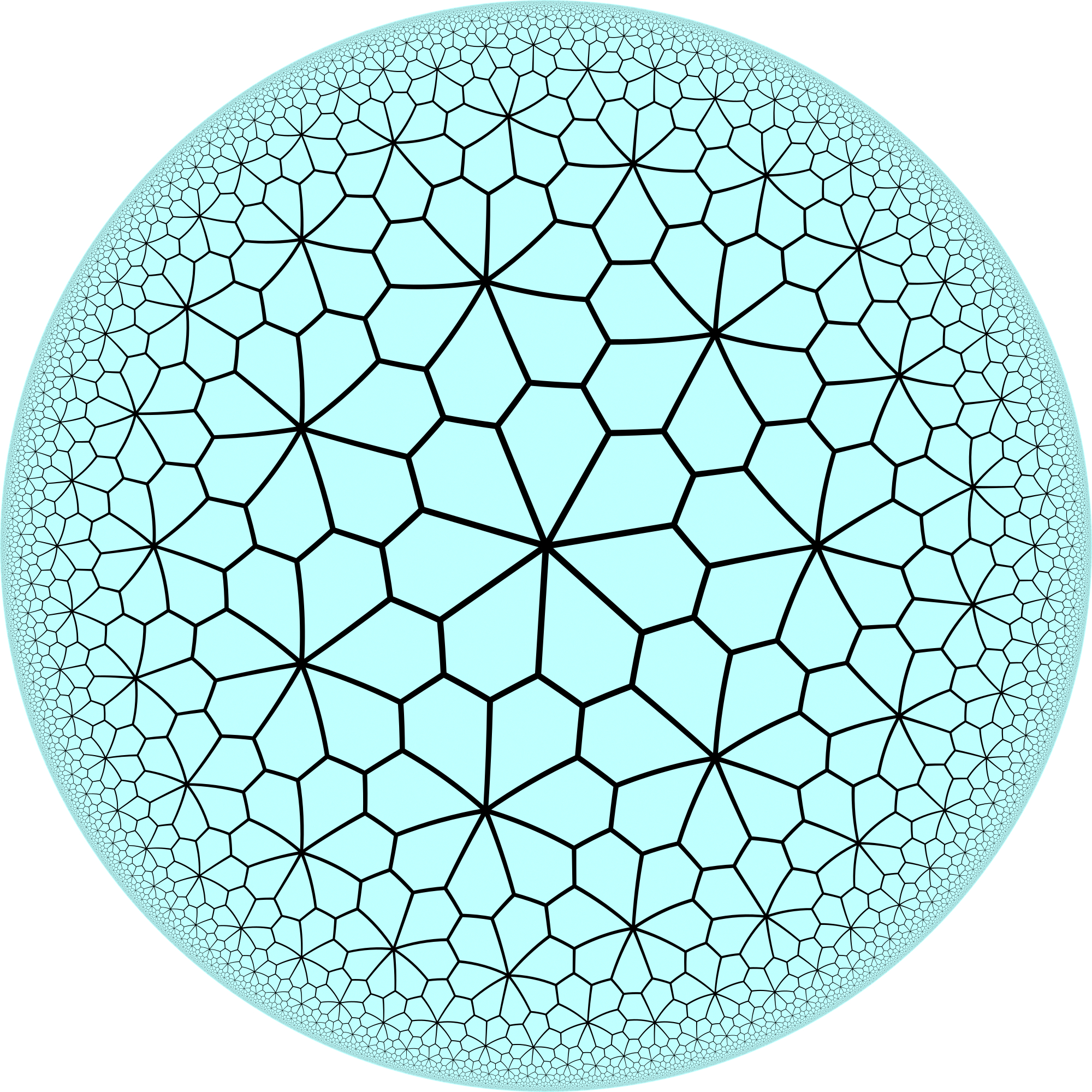
Poincaré disk model D of the hyperbolic plane, shown with pentagonal tiling to illustrate geodesics between embedded points. Source: https://commons.wikimedia.org/wiki/File:7-3_floret_pentagonal_tiling.svg, downloaded on 26 November 2024.

The geometric properties of PCA, extended to hPCA, allow for an insightful low-dimensional visualization method that highlights key features and dependencies of the input dataset. Tools from differential geometry applied to hPCA can be used to project the output of the local PCAs onto a low-dimensional hyperbolic manifold, the Poincaré disk D (Figure 1). This results in a two-dimensional display suitable for high-dimensional and large datasets, where major shared features of the data are shown prominently near the center of the disk, while less salient features are de-emphasized and displayed close to the edge.

The proposed hPCA visualization method is an extension of the canonical biplot used in PCA [12]. Biplots visualize data by projecting the input variables onto the first and second PCs. The resulting display is highly dependent on the ability of the two leading PCs to separate data points and is of limited use in high-dimensional datasets due to clutter and overplotting. The proposed hPCA visualization method uses the hyperbolic manifold $H^{k}$ to minimize these problems.

To motivate this approach, note that the data covariance matrix Σ and its diagonalization Λ are points on the high-dimensional smooth manifold GL(m,R). All local rotations constructed by hPCA are of the form $V_{l}^{T}\ldots V_{1}^{T}\Sigma V_{1}\ldots V_{l}$, and therefore are points on GL(m,R) as well. Each rotation matrix $V_{l}$ is from the embedded subgroup SO(k) of dimension $k(k-1)/2\ll m^{2}$, where k is the number of variables in the largest merger. The visualization method first orients the basis vectors of $V_{l}$ for each l and then projects onto the k-dimensional hyperboloid $H^{k}$. The individual mergers of the hPCA algorithm are identified with a sequence of points connected by geodesics on this smooth manifold. The points on $H^{k}$ are then mapped onto the two-dimensional Poincaré disk D or upper half plane U to obtain the final low-dimensional visualization.

The rootlets hPCA algorithm outputs a tree T with elements, or *branches*, consisting of the eigendecomposition of a local PCA. Each branch has a rotation matrix $V_{l}$ from SO(k), and associated eigenvalues $\lambda_{l}$ formatted as a vector from $R^{k}$, as well as indices corresponding to the input variables and the output leading PC of the local PCA. The visualization method uses $V_{l}$ and $\lambda_{l}$ from a branch of T to embed all hPCA variables into $H^{k}$.

The key step in the visualization method is the composite function $\varphi_{I,p}$ used to embed variable i in $H^{k}$ around point p using $V_{l}$ and $\lambda_{l}$ as input. The composite function $\varphi_{i,p}:SO(k)\times R^{k}\to H^{k}$ can be summarized as

(12) $$\varphi_{i,p}={exp}_{p}\circ \tau_{o\to p}\circ \iota_{0}\circ \Delta_{i}.$$

Equation (12) is constructed and described in greater detail in the Supplementary Materials. A brief overview is provided here, in order to clarify how the output of a local PCA is embedded as points on $H^{k}$. In Equation (12), $\Delta_{i}$ is the displacement coordinate function for variable $x_{i}$ as described in Appendix A, $\iota_{0}$ is the inclusion function that embeds vectors into the tangent space at the central point of $H^{k},\;\tau_{o\to p}$ is the parallel transport function between tangent spaces, and ${exp}_{p}$ is the exponential map function used to project vectors into $H^{k}$ around point p. A complete construction of $\varphi_{I,p}$, including an overview of relevant concepts from Riemannian and hyperbolic geometry, can be found in the Supplementary Materials.

Following embedding into $H^{k}$, the last step of the visualization algorithm maps the resulting point q in $H^{k}$ onto the two-dimensional Poincaré disk as the function $D:H^{k}\to D$

(13) $$D\left(q_{0},q_{1},\ldots ,q_{k}\right)=\left(\frac{q_{1},\;q_{2}}{q_{0}\;+\;1}\right).$$

The pseudo-code for the visualization algorithm is presented as Algorithm 2. The dimension k of $H^{k}$ is set to the dimension of the highest-dimensional local PCA (i.e., if the largest merger is between three input variables, k=3) in order to contain all orthogonal basis vectors. The algorithm then embeds the leading PC and input variables from the final branch constructed by the hPCA around o, using $\varphi_{I,p}$. Since the input variables are themselves leading PCs from previous local PCAs, Algorithm 2 recursively embeds all branches in a similar manner. Finally, the embedded points in $H^{k}$ are projected onto the Poincaré disk D or upper half plane U. After embedding, points are connected by paths along geodesics. If no clustering structure was found in the top level (i.e., $\lambda_{1}=\ldots =\lambda_{k}$ by ESET), paths are not plotted between points in the output cluster. If, in addition, no hierarchical structure was found by the rootlets algorithm (i.e., ESET null hypothesis accepted for all levels), input variables will be plotted as disconnected points embedded evenly spaced along the unit circle in D or U. Examples of output from Algorithm 2 are displayed in the next section.

**Algorithm 2:** Embedding of hPCA into the Poincaré disk.

1.	**Input:** Tree 𝒯 constructed by hPCA with B branches and indexing function T, dummy index $j_{B}$ for output of final branch in 𝒯, dimension k for $H^{k}$.	
2.	ℋ←{(1,0,…,0)}	# initialize list of points on $H^{k}$ with central point
3.	$\alpha \leftarrow \left\{j_{B}\right\}\;$	# initialize index set for ℋ with dummy index for final branch
4.	𝒟←Ø	# initialize list of points on Poincaré disk D
5.	**For** b←B,B-1,…,1 **Do**	
6.	(V,λ,ℐ,j)←T(b)	# get branch eigenvectors, values, input ind. set ℐ, output index j
7.	$i_{p}\leftarrow find\_ind(j,\alpha )$	# returns index of j in α
8.	$p\leftarrow H\left(i_{p}\right)$	# returns point p in ℋ indexed by $i_{p}$
9.	ℋ←{ℋ\{p}}	# remove point p from list
10.	$\alpha \leftarrow \left\{\alpha \backslash \left\{i_{p}\right\}\right\}$	# remove index for p from index set
11.	**For** i in ℐ **Do**	
12.	$q\leftarrow \phi_{i,p}(V,\lambda ,p)$	# project branch rotation for input i onto $H^{k}$
13.	d←D(q)	# map point onto D
14.	𝒟←𝒟∪{d}	
15.	ℋ←ℋ∪{q}	
16.	α←α∪{i}	
17.	**End For**	
18.	**End For**	
19.	**Return:** list of points D	

The computational complexity of Algorithm 2 depends on the total number of points embedded in $H^{k}$ and the cost of embedding a point with $\varphi_{I,p}$. Since $\varphi_{I,p}$ is a composite function, its complexity is the maximum of the individual functions in Equation (12). From the exact form of $\varphi_{I,p}$ detailed in the Supplementary Materials, each individual function involves elementary functions, vector addition, or calculating an inner product. These operations are on vectors of constant length, and therefore O(1) in all cases. The computational complexity of $\varphi_{I,p}$ is then O(1). If all hPCA branches are plotted, the total number of points embedded by Algorithm 2 is the maximum number of levels in Algorithm 1 as l=m-1. In this case, the computational complexity of Algorithm 2 is O(m).

## 3. Experiments

In this section, we introduce three example experiments to demonstrate the effectiveness and adaptability of the proposed hPCA framework. The results are compared against standard HCA algorithms [1] as implemented in SciPy (https://scipy.org/ (accessed on 26 November 2024)). Projections onto D or U were displayed using the Python library “hyperbolic” (https://pypi.org/project/hyperbolic/ (accessed on 26 November 2024)). Code and documentation to reproduce the experimental results are available at (https://github.com/koreywylie/rootlets_hPCA.git (accessed on 26 November 2024)).

### 3.1. Experiment 1

This experiment demonstrates that rootlets hPCA does not share a major limitation of HCA. HCA imposes a hierarchical structure on the input, regardless of whether or not such a structure is present in the data. The original treelets hPCA algorithm features the same limitation. In contrast, rootlets hPCA searches for clustering and hierarchical dependencies within the data but does not assume such structures exist. In fact, rootlets hPCA offers a statistical test for the existence of hierarchy within the data. Specifically, if the ESET null hypothesis is accepted during all levels of the rootlets algorithm as well as for the final output cluster, the data correlation matrix is the identity matrix by Theorem 1.

**Figure 2.**
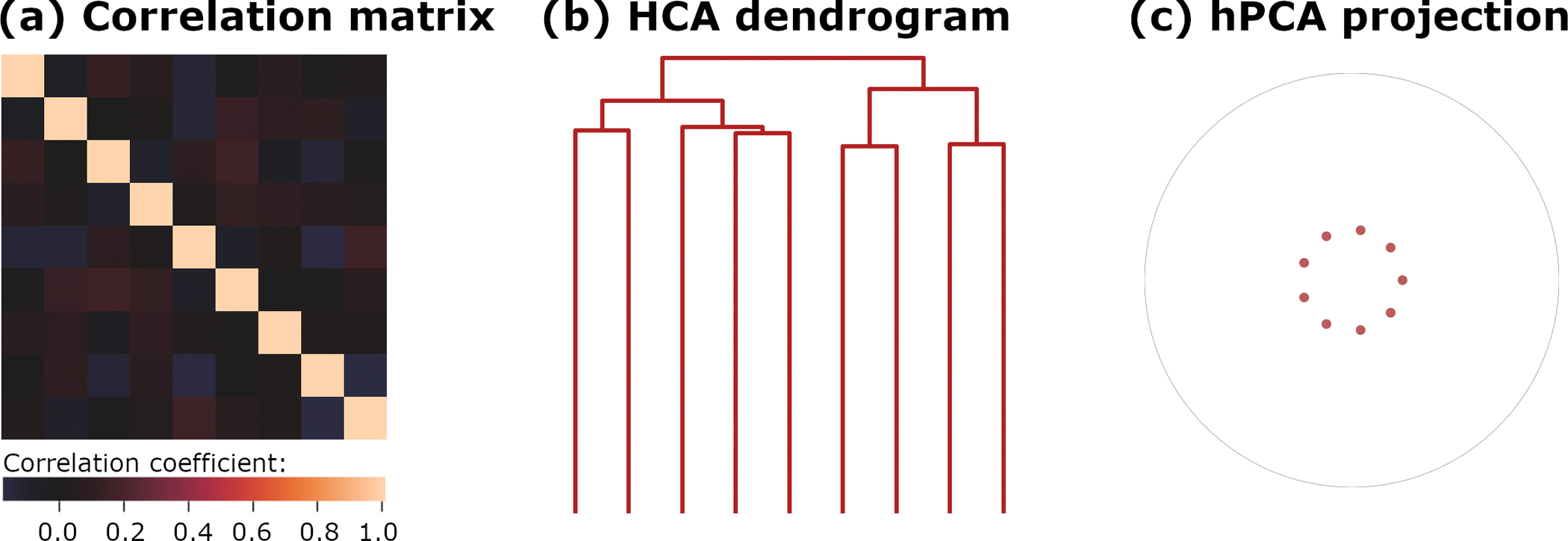
Correlation structure and analysis for Experiment 1. (**a**) Data correlation matrix. (**b**) Hierarchical clustering with Ward’s linkage. (**c**) hPCA results projected onto the Poincaré disk D. Since no hierarchical or clustering structure was found in the data, all variables input to hPCA are displayed as evenly spaced disconnected points.

To practically explore the effectiveness of rootlets hPCA, one hundred observations were drawn i.i.d. from nine Gaussian N(0,1) variables and entered into rootlets hPCA and HCA. In this experiment, there are no latent variables or hierarchical structure. For the rootlets algorithm, the *p*-value cutoff parameter was set to p=0.001/9=0.00011, where the denominator is the number of tests in a Bonferroni correction. The total number of tests is the number of hPCA levels (9 − 1 = 8 tests as ESET with p=1), plus one final test for the independence of variables in the output top level (ESET with p=0). HCA applied two agglomerative algorithms, Ward’s and single linkage. Results are displayed in Figure 2.

No hierarchical or clustering structure is evident in the sample correlation matrix (Figure 2a), with all off-diagonal correlations near 0 and ranging from −0.18 to 0.16. The hierarchy imposed by HCA on the input data can be seen in the dendrogram (Figure 2b). A nested subclustering structure is enforced on uncorrelated variables, appearing as paired mergers in the HCA dendrogram. Similarly, an artifactual hierarchy structure also resulted from applying an agglomerative single linkage HCA algorithm to the same dataset (Supplementary Figure S2). Additionally, a sequential series of mergers involving singleton variables is also visible in the single linkage dendrogram. This phenomenon, referred to as “chaining” [1], is another frequently observed defect of HCA algorithms.

Rootlets hPCA returned a single cluster of all input variables, without detecting any intermediary clusters. Furthermore, all eigenvalues in the final output cluster are equal by ESET $\chi^{2}(d.f.,\;N)=\chi^{2}(44,\;100)=29.987,\;p=0.947$ (uncorrected) = 1.0 (corrected). The results are then plotted on D as disconnected and evenly spaced points inside the disk. In this case, the lack of detail in the display is informative, as it reflects the nearly diagonal sample correlation matrix, and is consistent with the i.i.d. (or “spherical”) generative model.

In summary, in this experiment, HCA incorrectly imposed a hierarchy on the data as expected. In contrast, rootlets hPCA correctly concluded that the data was not hierarchically structured and that all variables are uncorrelated.

### 3.2. Experiment 2

Experiment 2 is a model of large-scale neural connectivity within the brain, as observed by functional magnetic resonance imaging (fMRI). Recent experimental evidence suggests neural processing systems are organized as a multiscale hierarchy, with increasingly specialized subsystems nested within more general processing systems [13–15]. The observed correlation matrix between regions of the brain or fMRI voxels (measured signal from localized points within the brain) features a nested block structure.

**Figure 3.**
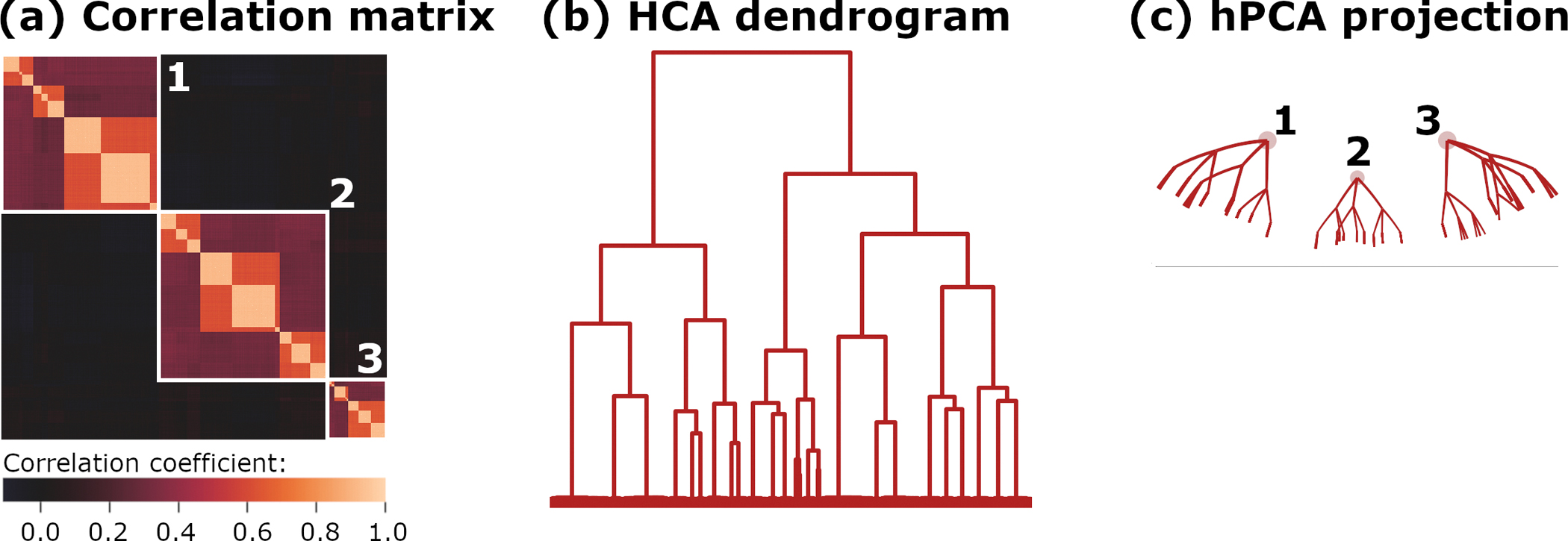
Correlation structure and analysis for Experiment 2. (**a**) Data correlation matrix, with large-scale structures labeled 1, 2, and 3 highlighted in white. (**b**) Hierarchical clustering with Ward’s linkage. (**c**) hPCA results projected onto the Poincaré upper-half plane U, with labels corresponding to (**a**).

A model designed to simulate this “networks-within-networks” structure has been described in detail [16]. Briefly, the experiment involves 1024 observed and 39 latent variables in a linear mixture model with added Gaussian noise. The latent variables are structured as 3-way subdivisions over 3 levels of a hierarchy. At each level, block sizes are drawn at random from a Dirichlet distribution with parameter alpha = (3, 3, 3). This resulted in blocks of variables corresponding to each latent variable, with sizes ranging from 2 to 448 observed variables. Time series for latent variables were constructed by drawing 1200 i.i.d. observations from a N(0,1) distribution. Gaussian noise was then added to all observed variables with an SNR of 2. The resulting correlation matrix is displayed in Figure 3a. For the rootlets algorithm, the *p*-value cutoff parameter was set to $p=0.001/1024=9.8\times 10^{-7}$ (Bonferroni corrected). Rootlets hPCA output was embedded into the Poincaré upper half plane U in Figure 3, since the vertical display facilitates comparison with the HCA dendrogram.

A nested block structure, consisting of 3-way subdivisions, is readily apparent in the sample correlation matrix, with blocks structured as in Equations (10) and (11). Blocks corresponding to the top-level latent variables are highlighted and labeled in Figure 3a. Correlations between these top-level blocks are nearly zero.

The HCA dendrogram incorrectly imposed a hierarchical structure upon the top-level latent variables, as shown in the top levels of Figure 3b. Although potential 3-way subdivisions are evident in some levels of the dendrogram (e.g., Figure 3b lower right branches), it is far from clear that this structure results from the underlying generative model of the data. Lastly, the lowest levels of the dendrogram are densely overplotted, forming a carpet without apparent distinctions between clusters.

The rootlets hPCA algorithm correctly identified the top-level latent variables as independent and uncorrelated (Figure 3c). These top-level variables as output from hPCA can be identified with large-scale features of the correlation matrix (Figure 3a), resulting in the labels shown in the display. The 3-way subdivisions of the generative data model can be seen throughout, although less apparent in places due to overlap in the low-dimensional projection. Additionally, overplotting is visible in the lowest levels of the hierarchy, corresponding to subdivisions of the smallest blocks in the correlation matrix into individual observed variables. However, even in this case, distinctions between individual blocks are preserved in the display.

Summarizing the above, HCA partially imposed a hierarchy that is not present in the data. The underlying generative model of the data was minimally reflected in the HCA dendrogram, and it is unclear which levels of the HCA dendrogram correspond to the levels of the true hierarchy. In contrast, rootlets hPCA correctly identified that the data are structured as three independent hierarchies, as well as fully reconstructed all levels. The major large-scale features of the data correlation matrix were identifiable in the rootlets hPCA display, but not the HCA dendrogram.

### 3.3. Experiment 3

**Figure 4.**
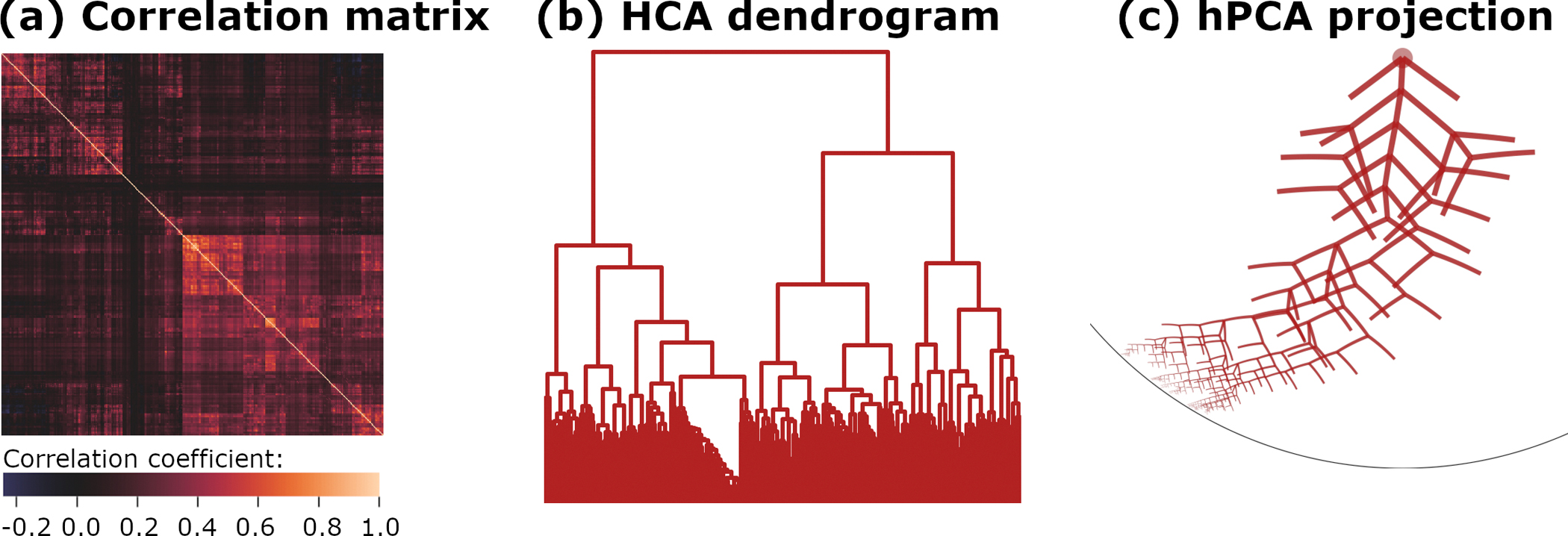
Correlation structure and analysis for Experiment 3. (**a**) Data correlation matrix. (**b**) Hierarchical clustering with Ward’s linkage. (**c**) hPCA results projected onto the Poincaré disk D.

This experiment applied the rootlets algorithm to analyze a neuroimaging dataset, the young adult Human Connectome Project (HCP, https://www.humanconnectome.org (accessed on 30 October 2024)). In this dataset, 1003 subjects were scanned using fMRI across 4 sessions of 15 min each, with a temporal sampling frequency of 1.39 Hz (MRI repetition time TR = 0.72 s). All subjects’ data were spatially and temporally aligned and compressed using a group-level PCA, as described in the HCP S1200 Extensively Processed data release (https://www.humanconnectome.org/study/hcp-young-adult (accessed on 30 October 2024)). Cortical data from 360 multimodal regions were then extracted by signal averaging [17]. The rootlets algorithm p-value cutoff parameter was set to $p=0.001/360=2.8\times 10^{-6}$ (Bonferroni corrected). Results are displayed in Figure 4.

Since our understanding of the human brain’s “networks-within-networks” structure is incomplete, it is unknown if the HCA dendrogram or the hPCA projection onto D better reflects the underlying biology. The HCA dendrogram’s binary mergers at each level are an artifact of the algorithm, however, and cannot be interpreted. In contrast, the single hierarchy constructed by rootlets hPCA encompassed all input variables. This is unlike the independent top-level clusters in Experiments 1 and 2, as can be seen by comparing Figure 4c with Figures 2c and 3c. Progressively lowering the rootlets p-value cutoff parameter increasingly grouped the top levels of the hierarchy into a single cluster, while leaving the arrangement of the lowest branches relatively unchanged (Supplementary Figure S3). This suggests the rootlets algorithm is uncertain on the structure of the top-level clusters, which involve minimally correlated variables. Resolving this issue will likely require a detailed comparison with the current understanding of the known biology of large-scale neuroimaging networks and is a topic for future investigation.

## 4. Conclusions

In this work, we propose the novel rootlets hPCA algorithm and data visualization method. Rootlets hPCA groups variables into clusters and constructs adaptive multiway mergers using statistical criteria, without imposing a hierarchy on non-hierarchical data. Spectral analysis demonstrates that rootlets hPCA clusters correspond to nested blocks of similar variables in the data correlation matrix. Tools from Riemannian geometry are then used to map hPCA output onto a compact low-dimensional hyperbolic manifold, the Poincaré disk. Experimental results on simulated data confirm that, in contrast to HCA, rootlets hPCA does not impose a hierarchy and can successfully identify i.i.d. variables. Furthermore, additional experiments suggest that rootlets hPCA is able to fully reconstruct hierarchies with random mergers, and is potentially useful for analyzing large data sets with unknown hierarchical structure.

An important additional feature of hPCA algorithms is their ability to accurately estimate latent variables in data [16]. Since the main focus of this work is a comparison with HCA, and since HCA does not estimate latent variables, this fundamental ability of hPCA falls outside the scope of the current work. Notably, in any applied data science field, identifying and characterizing the major individual features of the data (i.e., latent variables or leading PCs) is often the primary goal of the analysis. This additional feature is a major strength of hPCA algorithms.

An additional important aspect of future work on hPCA will be incorporating more advanced embedding techniques for dimensionality reduction from $H^{k}$ onto D, rather than projection onto the first two coordinates as in the current visualization method. Recently, hyperbolic variational autoencoders (VAE) [18,19] or t-stochastic neighborhood embedding (t-SNE) [20] have been developed. Additionally, hPCA could be improved using distributed learning algorithms such as non-convex optimization [21] to allow for parallelized computation and the estimation of time-varying network structures. This extension of rootlets hPCA would likely require optimizing an objective function similar to Equation (3), however, over a large number of potentially non-sparse rotation matrices. We hope that future work will evaluate and incorporate these methods and continue to expand the hPCA framework.

## Supplementary Material

Brief Overview of Riemannian Geometry

Figure S2: Single linkage Hierarchical Clustering Analysis for Experiment 1

Figure S3: Rootlets hPCA p-value cutoff parameter comparison

## Data Availability

The data that support the findings of this study are available from the Human Connectome Project, https://www.humanconnectome.org/ (accessed on 30 October 2024). These data are publicly available to researchers who agree to the data use terms.

## Appendix A

The sequences of local PCAs constructed by hPCA algorithms have natural geometric interpretations that can be used to construct a visualization of the dependency structure of the data. A key step in this process is embedding the input data points and resulting PCs within a common Euclidean space and calculating distances between their respective coordinates.

In a k-dimensional PCA, the eigenvectors form an orthonormal basis for $R^{k}$. Distances and angles between PCs and data points are calculated by embedding vectors within the subspace ${\left\{w_{j}\right\}}_{j=1,\ldots ,k}$ in $R^{k}$ using the singular value decomposition (SVD) of the data,

(A1)
$$X=V\Lambda^{1/2}W^{T},$$

where $W=\left[w_{1},\ldots ,w_{k}\right]$ is the matrix of orthonormal right singular vectors $w_{j}$.

Using the SVD, the local coordinates of the PCs $u_{j}$ within ${\left\{w_{j}\right\}}_{j=1,\ldots ,k}$ can be represented as

(A2)
$$\begin{array}{c} U=V^{T}X=V^{T}V\Lambda^{1/2}W^{T}=\Lambda^{1/2}W^{T},\\ \left[\begin{array}{c} u_{1}^{T}\\ \vdots \\ u_{k}^{T} \end{array}\right]=\left[\begin{array}{ccc} \sqrt{\lambda_{1}} & \cdots & 0\\ \vdots & \ddots & \vdots \\ 0 & \cdots & \sqrt{\lambda_{k}} \end{array}\right]\left[\begin{array}{c} w_{1}^{T}\\ \vdots \\ w_{k}^{T} \end{array}\right]\\ u_{j}=\sqrt{\lambda_{j}}w_{j},\;j=1,\ldots ,k. \end{array}$$

The PCs are thus scaled versions of the right singular vectors, with lengths equal to the square root of the corresponding eigenvalues. Their coordinates within the subspace are simply these lengths along the corresponding basis vector $w_{j}$. For instance, the local coordinates for PC_1_ are calculated as $\left\langle u_{1},w_{j}\right\rangle$, with resulting local coordinates $\lambda_{j}^{½}$ for j=1 and 0 otherwise.

The local coordinates of the data points $x_{i}$ within ${\left\{w_{j}\right\}}_{j=1,\ldots ,k}$ are determined in a similar manner. To simplify notation, let $Z=V\Lambda^{1/2}=[z_{ij}]=\left[\lambda_{j}^{½}v_{ij}\right]$ for i=1,…,m and j=1,…,k, where $v_{ij}$ is the i th element of eigenvector $v_{j}$. The data matrix X can then be written as

(A3)
$$\begin{array}{c} X=V\Lambda^{1/2}W^{T}=ZW^{T},\\ \left[\begin{array}{c} x_{1}^{T}\\ x_{2}^{T}\\ \vdots \\ x_{m}^{T} \end{array}\right]=\left[\begin{array}{cccc} z_{11} & z_{12} & \cdots & z_{1k}\\ z_{21} & z_{22} & \cdots & z_{2k}\\ \vdots & \vdots & \ddots & \vdots \\ z_{m1} & z_{m2} & \cdots & z_{mk} \end{array}\right]\left[\begin{array}{c} w_{1}^{T}\\ w_{2}^{T}\\ \vdots \\ w_{k}^{T} \end{array}\right],\\ x_{i}=\sum_{j=1}^{k}\;z_{ij}w_{j}=\sum_{j=1}^{k}\;\sqrt{\lambda_{j}}v_{ij}w_{j},\;i=1,\ldots ,m. \end{array}$$

The local coordinates of a data point within ${\left\{w_{j}\right\}}_{j=1,\ldots ,k}$ are then determined using the final expression in Equation (A3). For instance, the k coordinates for data point $x_{i}$ are $\lambda_{j}^{½}v_{ij}$ for j=1,…,k. With the PCs and data points embedded within the same Euclidean coordinate system, the displacement vector between the leading PC $u_{1}$ and each data point can be calculated by taking the difference of Equations (A2) and (A3). Since this calculation involves elements of the eigenvector matrix $V=\left[v_{ij}\right]$ and eigenvalue vector λ, it can be summarized as the displacement coordinate function $\Delta_{i}:SO(k)\times R^{k}\to R^{k}$,

(A4)
$$\Delta_{i}\left(V,\lambda \right)=x_{i}-u_{1}=\left(\begin{array}{cccc} \sqrt{\lambda_{1}}\left(v_{i1}-1\right), & \sqrt{\lambda_{2}}v_{i2}, & \cdots , & \sqrt{\lambda_{k}}v_{ik} \end{array}\right),$$

with i indexing the data point $x_{i}$.

## Appendix B

Proposition 1 is proved by considering the eigenvalue sequence of all possible mergers constructed by hPCA and how the branching process affects the eigenvalue sequences. Mergers with singleton variables are possible; however, the proposition does not apply in this case since k=1 for these clusters. Notably, in order to cover all possible scenarios, the induction step will need to consider mergers involving a singleton cluster. Such mergers are possible under hPCA algorithms and will result in a cluster of size k≥2.

### Proof of Proposition 1.

The proposition is proved using an induction argument. The domain is all positive integers k≥2. The base case is k=2. The induction hypothesis is that, for each pair of clusters merged, the pre-merge eigenvalue sequences feature one or two distinct eigenvalues. The induction step will be divided into two cases, depending on the cluster defining criteria applied by the rootlets algorithm.

If k=2, then $\lambda_{1}\geq \lambda_{2}$, a sequence with one (if $\lambda_{1}=\lambda_{2}$) or two distinct values (if $\lambda_{1}>\lambda_{2}$). The base case is therefore true for all clusters of size k=2.

In the induction step, the rootlets algorithm considers merging two clusters of sizes a and b, with a combined size a+b=k. The case of a=b=1 was proved as the base case of k=2 above. For k≥3, the induction step will be proved in two cases, from the types of mergers constructed by the rootlets algorithm. Case 1 involves simple mergers, under the cluster defining criteria that $\lambda_{2}=\ldots =\lambda_{k}$ for the combined cluster. Case 2 involves branch mergers, when $\lambda_{i}>\lambda_{j}$ for some i and j in 2≤i<j≤k in the potential combined cluster.

Case 1 (simple merger): If the cluster defining criteria is true, then $\lambda_{2}=\ldots =\lambda_{k}$ for the combined cluster, and the elements of each cluster are merged by the algorithm to form a single larger cluster of size k satisfying the cluster defining criteria. Since $\lambda_{1}\geq \lambda_{2}=\ldots =\lambda_{k}$ in this case, the proposition is true.

Case 2 (branch merger): When a cluster is branched, its elements are first fixed in their pre-merge state, including the pre-merge eigenvalue sequences, and a new cluster is created from the leading PCs. It is therefore sufficient to show that the proposition is true for both clusters in their pre-merge state, as well as that the proposition is true for the new cluster. The proposition will need to be proved for a merger involving a singleton cluster of size a=1 or b=1, before continuing with mergers of clusters of sizes a,b≥2.

Let a=1 and b≥2. Prior to a branch merger, the proposition does not apply to a singleton cluster of size a=1, and the proposition is true by the induction hypothesis for a cluster of size b≥2. The proof for a≥2 and b=1 is similar. If both a≥2 and b≥2, the proposition is true for both clusters in their pre-merge states by the induction hypothesis.

After branching, a new cluster is created of size k=2 from the leading PCs. The eigenvalue sequence of the new cluster is $\lambda_{1}\geq \lambda_{2}$. The proposition is therefore true for the new cluster, finishing the proof of the induction step. □

## Appendix C

### Proof of Lemma 1.

Eigenvectors $v_{2},\ldots ,v_{k}$ of Σ are k-1 linearly independent solutions of (Σ-μI)v=0, where μ is the eigenvalue associated with general solution v. In the following, μ considered as an element of R will be used, but μ may or may not equal $\lambda_{1}$ or the repeated eigenvalue $\lambda =\lambda_{2}=\ldots =\lambda_{k}$.

If $\lambda_{1}>\lambda$, no other linearly independent solution exists, while if $\lambda_{1}=\lambda_{2}$, eigenvector $v_{1}$ is the only other linearly independent solution. To show this, let v be a linear combination of all eigenvectors,

(A5)
$$v=\sum_{i=1}^{k}x_{i}v_{i},\;x_{i}\in R.$$

If v is a solution to (Σ-μI)v=0, then μv=Σv, and

(A6)
$$\mu v=\mu \left(\sum_{i=1}^{k}\;x_{i}v_{i}\right)=\Sigma \left(\sum_{i=1}^{k}\;x_{i}v_{i}\right)=\sum_{i=1}^{k}x_{i}\Sigma v_{i}=\sum_{i=1}^{k}x_{i}\lambda_{i}v_{i}.$$

If $\lambda_{1}>\lambda$, then since $\mu x_{i}=\lambda_{i}x_{i}$ for all i=1,…,k by the above calculation, $\lambda_{1}x_{1}=\mu x_{1}$ implies $x_{1}=0$ and v is linearly dependent on eigenvectors $v_{2},\ldots ,v_{k}$. If $\lambda_{1}=\lambda_{2}=\lambda$, a similar calculation shows that there are k linearly independent solutions of (Σ-μI)v=0=(Σ-λI)v.

Let Ψ=Σ-λI be a symmetric k-by-k matrix. The rank of Ψ is calculated as rank(Ψ)=dim(Image(Ψ))-dim(Kernel(Ψ)). The dimension of the image of Ψ is k. The kernel of Ψ is defined as Ψv=(Σ-λI)v=0. If $\lambda_{1}>\lambda$, then dim(Kernel(Ψ))=k-1 by the above calculation. The rank of Ψ is then rank(Ψ)=k-(k-1)=1. If $\lambda_{1}=\lambda$, then dim(Kernel(Ψ))=k and rank(Ψ)=k-(k)=0,implyingΨ=0.

Finally, if v is an eigenvector of Ψ with eigenvalue v, then v is an eigenvector of Σ with eigenvalue (v+λ) as shown by

(A7)
Σv=(Ψ+λI)v=Ψv+λv=vv+λv=(v+λ)v.

□

## Appendix D

### Proof of Lemma 2.

If $\lambda_{1}>\lambda_{2},\;\Psi$ is a matrix of rank 1 by Lemma 1. Let $\Psi =vvv^{T}$, where v is a unit length k-vector and v is a scalar. By the proof of Lemma 1, v≠0. Since $\Psi v=vvv^{T}v=vv,\;v$ is an eigenvector of Ψ with eigenvalue v. Since Ψ is a positive definite matrix of rank 1, v is the only non-zero eigenvalue and v>0. By Lemma 1, $\lambda_{1}=(v+\lambda )$ and $\lambda =\lambda_{2}=\ldots =\lambda_{k}$ are the only eigenvalues of Σ. By rearranging the terms, $v=\lambda_{1}-\lambda$.

An expression for v independent of $\lambda_{1}$ but dependent on k, is found using the trace of Σ. By hypothesis, $\Sigma_{ii}=1$ for i=1,…,k, and

(A8)
$$\begin{array}{c} tr\left(\Sigma \right)=\sum_{i=1}^{k}\;\Sigma_{ii}=k=\sum_{i=1}^{k}\;\lambda_{i},\\ k=\lambda_{1}+(k-1)\lambda =(v+\lambda )+(k-1)\lambda ,\\ v=k(1-\lambda ). \end{array}$$

The elements $v_{i}$ of v are then calculated from the diagonal elements $\Sigma_{ii}$ of Σ using Lemma 1,

(A9)
$$\begin{array}{c} \Sigma =\Psi +\lambda I=vvv^{T}+\lambda I=v\left[\begin{array}{cccc} v_{1}^{2} & v_{1}v_{2} & \cdots & v_{1}v_{k}\\ v_{2}v_{1} & v_{2}^{2} & \cdots & v_{2}v_{k}\\ \vdots & \vdots & \ddots & \vdots \\ v_{k}v_{1} & v_{k}v_{2} & \cdots & v_{k}^{2} \end{array}\right]+\left[\begin{array}{cccc} \lambda & 0 & \cdots & 0\\ 0 & \lambda & \cdots & 0\\ \vdots & \vdots & \ddots & \vdots \\ 0 & 0 & \cdots & \lambda \end{array}\right],\\ \Sigma_{ii}=1=vv_{i}^{2}+\lambda ,i=1,\ldots ,k,\\ v_{i}=\pm \sqrt{\frac{1-\lambda }{v}}=\pm \sqrt{\frac{\left(1-\lambda \right)}{k(1-\lambda )}}=\pm \frac{1}{\sqrt{k}}, \end{array}$$

where the last line results from solving for $v_{i}$ in the middle line and substituting v from Equation (A8).

If $\lambda_{1}=\lambda_{2}=\lambda ,\;\Psi =0$ is a matrix of zeros by Lemma 1 and Σ=λI. Since $\Sigma_{ii}=1$ for all i by hypothesis, $\lambda =\Sigma_{ii}=1$, and Σ=I. □

## Appendix E

### Proof of Theorem 1.

If $\lambda_{1}>\lambda_{2}$, by Lemma 1 and Equation (A9), the elements of Σ, expressed as elements of the eigenvector v and the scalar v, are

(A10)
$$\Sigma =\left[\begin{array}{cccc} 1 & vv_{1}v_{2} & \cdots & vv_{1}v_{k}\\ vv_{2}v_{1} & 1 & \cdots & vv_{2}v_{k}\\ \vdots & \vdots & \ddots & \vdots \\ vv_{k}v_{1} & vv_{k}v_{2} & \cdots & 1 \end{array}\right].$$

Using the decomposition by X, the elements of Σ are

(A11)
$$\Sigma =\frac{XX^{T}}{n-1}=\frac{1}{n-1}\left[\begin{array}{c} x_{1}^{T}\\ \vdots \\ x_{k}^{T} \end{array}\right]\left[\begin{array}{lll} x_{1} & \cdots & x_{k} \end{array}\right]=\frac{1}{n-1}\left[\begin{array}{cccc} x_{1}^{T}x_{1} & x_{1}^{T}x_{2} & \cdots & x_{1}^{T}x_{k}\\ x_{2}^{T}x_{1} & x_{2}^{T}x_{2} & \cdots & x_{2}^{T}x_{k}\\ \vdots & \vdots & \ddots & \vdots \\ x_{k}^{T}x_{1} & x_{k}^{T}x_{2} & \cdots & x_{k}^{T}x_{k} \end{array}\right].$$

Equating the off-diagonal elements of Equations (A10) and (A11),

(A12)
$$\frac{x_{i}^{T}x_{j}}{n-1}=vv_{i}v_{j},\;\mathrm{for}\;1\leq i,j\leq k,i\neq j.$$

By hypothesis, $\left\langle x_{i},x_{j}\right\rangle =x_{i}^{T}x_{j}\geq 0$. Since $v=\lambda_{1}-\lambda$ by Lemma 2, v>0, and ${x_{i}}^{T}x_{j}\geq 0$ then implies $v_{i}v_{j}\geq 0$ for all i,j≤k. This can only be true if $sign\left(v_{i}\right)=sign\left(v_{j}\right)$ is a constant for all i,j, where sign(⋅) is the signum function. Let $s=sign\left(v_{i}\right)$. Since $v_{i}\neq 0$ by Equation (A9), s≠0. Using Equation (A9), eigenvector v and the general form of Σ are then

(A13)
$$\begin{array}{c} v=\frac{s}{\sqrt{k}}1,\\ \Sigma =vvv^{T}+\lambda I=\frac{v}{k}11^{T}+\lambda I. \end{array}$$

To complete the proof for $\lambda_{1}>\lambda_{2}$, it remains to show that v/k=r is the difference in eigenvalues of Σ scaled to k, and that λ=(1-r). By Lemma 2, $v=\lambda_{1}-\lambda$, and $r=v/k=\left(\lambda_{1}-\lambda \right)/k$. Lastly, λ=(1-v/k)=(1-r) follows from solving for λ in v=k(1-λ) from Equation (A8).

Finally, if $\lambda_{1}=\lambda_{2}$, then Σ=I by Lemma 2 and $\Sigma =r11^{T}+(1-r)I=I$ is true if and only if r=0. □

## Appendix F

### Proof of Theorem 2.

If r=0, then R=I and $\lambda_{1}=\ldots =\lambda_{k}=1$ by the characteristic function of the identity matrix.

If r>0, by a direct calculation $k^{\left(-1/2\right)}1$ is an eigenvector of R,

(A14)
$$\begin{array}{c} R\left(\frac{1}{\sqrt{k}}1\right)=r11^{T}\left(\frac{1}{\sqrt{k}}1\right)+(1-r)\left(\frac{1}{\sqrt{k}}1\right)=rk\left(\frac{1}{\sqrt{k}}1\right)+(1-r)\left(\frac{1}{\sqrt{k}}1\right),\\ R\left(\frac{1}{\sqrt{k}}1\right)=((k-1)r+1)\left(\frac{1}{\sqrt{k}}1\right), \end{array}$$

since $1^{T}1=k$. From the above, the corresponding eigenvalue is μ=(k-1)r+1.

Let λ≠μ be another eigenvalue of R with associated eigenvector v and let 0 be a k-vector of zeros. Since λ≠μ,1 is a scaled eigenvector different from v;v is then orthogonal to 1 and $1^{T}v=0$. Relative to parameter r,λ is then obtained by direct calculation,

(A15)
$$\begin{array}{c} \lambda v=Rv=r11^{T}v+(1-r)v,\\ (\lambda -1+r)v=r11^{T}v=0,\\ (\lambda -1+r)=0,\\ \lambda =1-r. \end{array}$$

Since the rank of R is k and since μ is an eigenvalue of R different from λ, the above holds for k-1 eigenvectors and λ is a repeated eigenvalue of multiplicity k-1.

It remains to be shown that μ is the leading eigenvector $\lambda_{1}$. Since r>0 and k>0,

(A16)
μ=(k-1)r+1=kr-r+1>1-r=λ.

Eigenvalue μ is then greater than all other eigenvalues and $\mu =\lambda_{1}$. □

## Appendix G

### Proof of Theorem 3.

Let X be the m-by-n concatenated matrix with rows $x_{i}$ such that

(A17)
$$\frac{1}{n-1}XX^{T}=\frac{1}{n-1}\left[\begin{array}{l} X_{a}\\ X_{b} \end{array}\right]{\left[\begin{array}{l} X_{a}\\ X_{b} \end{array}\right]}^{T}=\left[\begin{array}{cc} R_{a} & R_{ab}\\ R_{ab}^{T} & R_{b} \end{array}\right],$$

so that m=a+b is the number of rows in X and a+1 indexes the first row of $X_{b}$ within X.

Let $v_{1a},\;v_{1b}$ be the leading eigenvectors of $R_{a}$ and $R_{b}$, respectively. By Lemma 2, $v_{1a}$ and $v_{1b}$ are both scaled one-vectors of lengths a and b, respectively, with scaling factors equal to their lengths. The leading PCs $u_{1a},\;u_{1b}$ are calculated by projecting the matrices $X_{a}$ and $X_{b}$ onto $v_{1a}$ and $v_{1b}$,

(A18)
$$\begin{array}{c} u_{1a}={\left(v_{1a}^{T}X_{a}=\frac{1}{\sqrt{a}}\left[\begin{array}{lll} 1 & \cdots & 1 \end{array}\right]\left[\begin{array}{c} x_{1}^{T}\\ \vdots \\ x_{a}^{T} \end{array}\right]\right)}^{T}=\frac{1}{\sqrt{a}}\sum_{h=1}^{a}\;x_{h},\\ u_{1b}={\left(v_{1b}^{T}X_{b}=\frac{1}{\sqrt{b}}\left[\begin{array}{lll} 1 & \cdots & 1 \end{array}\right]\left[\begin{array}{c} x_{a+1}^{T}\\ \vdots \\ x_{m}^{T} \end{array}\right]\right)}^{T}=\frac{1}{\sqrt{b}}\sum_{i=a+1}^{m}\;x_{i}. \end{array}$$

The inner product of vectors $u_{1a}$ and $u_{1b}$ is calculated according to

(A19)
$$\left\langle u_{1a},u_{1b}\right\rangle =u_{1a}^{T}u_{1b}={\left(\frac{1}{\sqrt{a}}\sum_{h=1}^{a}\;x_{h}\right)}^{T}\left(\frac{1}{\sqrt{b}}\sum_{i=a+1}^{m}\;x_{i}\right)=\frac{1}{\sqrt{ab}}\sum_{h=1}^{a}\sum_{i=a+1}^{m}x_{h}^{T}x_{i}.$$

The elements of $R_{ab}$ and their average are calculated as

(A20)
$$\begin{array}{c} R_{ab}=\frac{X_{a}X_{b}^{T}}{n-1}=\frac{1}{n-1}\left[\begin{array}{c} x_{1}^{T}\\ \vdots \\ x_{a}^{T} \end{array}\right]\left[\begin{array}{lll} x_{a+1} & \cdots & x_{m} \end{array}\right]=\frac{1}{n-1}\left[\begin{array}{ccc} x_{1}^{T}x_{a+1} & \cdots & x_{1}^{T}x_{m}\\ \vdots & \ddots & \vdots \\ x_{a}^{T}x_{a+1} & \cdots & x_{a}^{T}x_{m} \end{array}\right],\\ \frac{1}{ab}sum\left(R_{ab}\right)=\frac{1}{ab(n-1)}\sum_{h=1}^{a}\;\sum_{i=a+1}^{m}\;x_{h}^{T}x_{i}=\frac{1}{ab(n-1)}\sqrt{ab}\left\langle u_{1a},u_{1b}\right\rangle , \end{array}$$

where sum(⋅) indicates element-wise summation. Summarizing the above with $1_{c}$ denoting a c-vector of ones,

(A21)
$$\begin{array}{c} \frac{\left\langle u_{1a},u_{1b}\right\rangle }{\left(n-1\right)}=\frac{1_{a}^{T}R_{ab}1_{b}}{\sqrt{ab}},\\ \frac{\left\langle u_{1a},u_{1b}\right\rangle }{\sqrt{ab}(n-1)}=\frac{1_{a}^{T}R_{ab}1_{b}}{ab}=\frac{1}{ab}sum\left(R_{ab}\right). \end{array}$$

□
