# Supplementary material for: Rootlets Hierarchical Principal Component Analysis for Revealing Nested Dependencies in Hierarchical Data
Source: Mathematics (Basel). Author manuscript; Available in PMC 2025 Sep 24. (PMC12456745; doi:10.3390/math13010072)
Supplement: Brief Overview of Riemannian Geometry [file NIHMS2060439-supplement-Brief_Overview_of_Riemannian_Geometry.docx]

**Supplementary Materials**

The purpose of this supplement is to provide a more detailed background and systematic development of the hPCA visualization method from section 2.5 in the main text. In keeping with the assumed linear algebra background of the main text, the materials in this supplement are focused on understanding the fundamental concepts needed to apply Riemannian geometry, rather than rigorous derivations of the relevant equations.

Section S1 qualitatively describes how overlapping local coordinate systems can be combined in order to navigate on curved spaces. Section S2 provides a brief overview of relevant concepts from differential geometry. Section S3 provides an overview of hyperbolic geometry, including models of hyperbolic space and the ambient spaces they are embedded within. Section S4 develops the machinery from Riemannian geometry needed to work in hyperbolic spaces.

Notation in this section is standard for differential geometry. Points on a manifold will be denoted with italics as *p*, *q* with coordinate representations ***p***, ***q***. Non-Euclidean functions will be distinguished from their Euclidean equivalents using subscripts (e.g., ⟨·,·⟩*_x_* and ||·||*_x_*).

**S1. Local PCAs and Local Coordinate Charts**

A series of local coordinate systems that overlap at well-defined points, in combination with smooth mapping functions between respective coordinates, are referred to as *charts*. Using their shared points, overlapping charts can be glued together and projected onto an underlying curved surface. For example, in the field of cartography, overlapping flat maps are aligned and projected onto a sphere, creating a globe that displays the entire surface of the earth. Notably, in this example, while individual flat maps are in terms of Euclidean coordinates, the curved surface of the earth is non-Euclidean.

In the current work, hPCA algorithms are a series of local PCAs with a local Euclidean coordinate system constructed at every level. Furthermore, since the output of the local PCA from a lower level is input to a subsequent local PCA at a higher level, these coordinate systems overlap at specific points. Within each local coordinate system, distances and displacement vectors between points are calculated as in Appendix A. These distances are mapped to an underlying curved manifold, such as the hyperbolic space ℍ*^n^*, using the tools of differential geometry.

**S2. Differential Geometry**

Precise measurements on curved manifolds requires concepts and methods from differential geometry. Concepts relevant to the current work are highlighted below. Specific examples involving hyperbolic geometry will be provided in section S3. For an in-depth introduction to differential geometry, specifically for definitions without requiring an ambient space, we refer the reader to the texts by Lee [22,23] in the references.

A *manifold* M of dimension *n* is a set that can be locally approximated by the Euclidean space ℝ*^n^*. For example, the surface of a sphere embedded in ℝ^3^ can be locally approximated by the flat plane ℝ^2^ and is therefore a 2-dimensional manifold. In this example, ℝ^3^ is the ambient space in which the manifold is embedded.

The *tangent space* *T_p_*M at point *p* from M is the (*n*+1)-dimensional vector space approximating M around *p* (Figure S1, left). Using the inner product from the ambient space, *T_p_*M can be defined using an orthogonality condition. For instance, if the ambient space is Euclidean, vector ***u*** is an element of *T_p_*M if ⟨***u***, ***p***⟩ = 0 where ***p*** is the coordinate representation of *p*. Note that *T_p_*M ≠ *T_q_*M if *p* ≠ *q*.
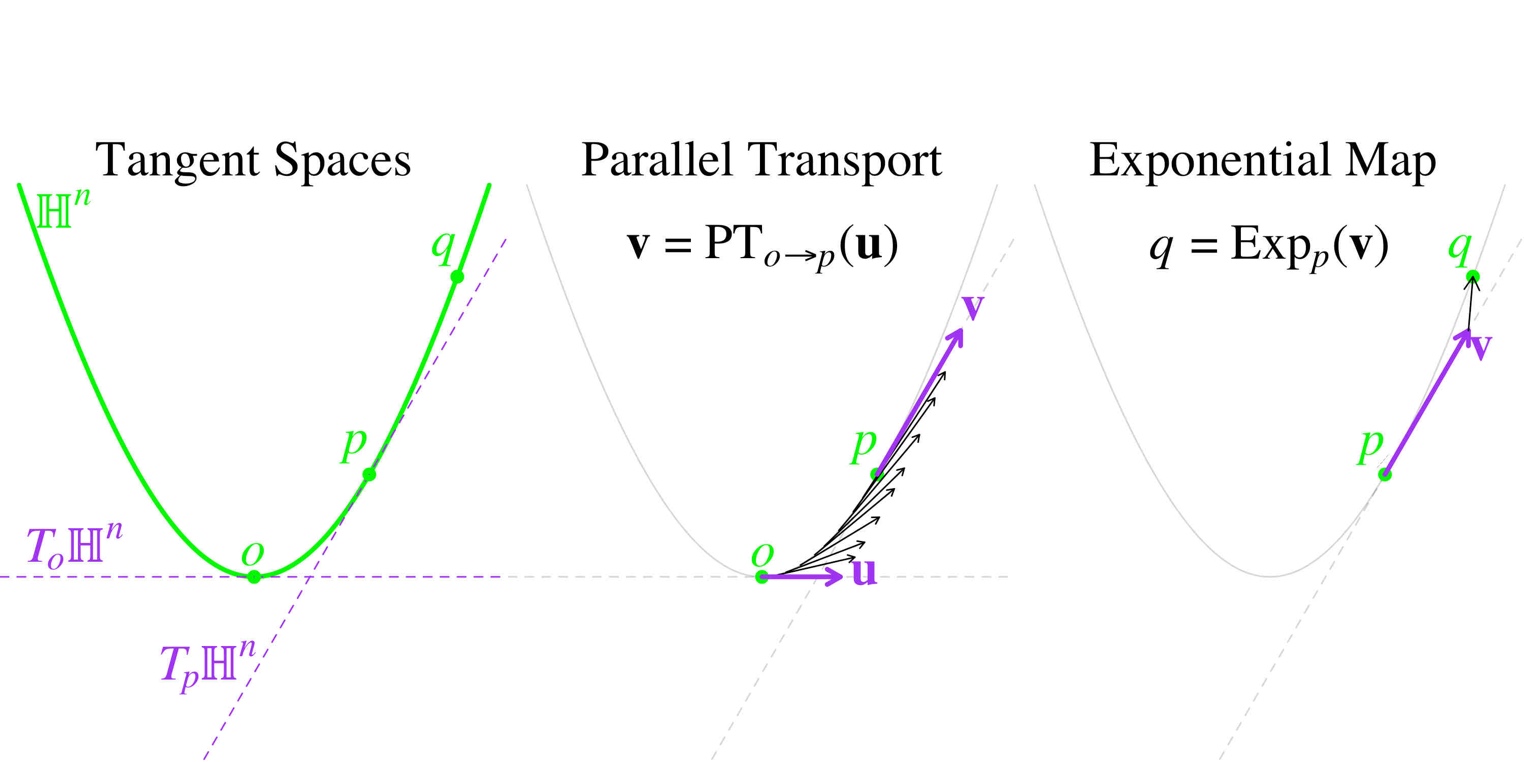


Figure S1: Riemannian geometry on a hyperbolic manifold

A *Riemannian metric* *g_p_* : *T_p_*M x *T_p_*M 🡪 ℝ is a collection of inner products on each *T_p_*M that vary smoothly depending on point *p*. It defines the local geometry of M at each point and is used in calculating the lengths of curves on the manifold and distances between points on M. If ***G*** = *g_p_* is the matrix form of the Riemannian metric at point *p***,** the inner product ⟨·,·⟩*_p_* and norm ||·||*_p_* are calculated as ⟨***u***,***v***⟩*_p_* = ***uGv****^T^* and ||***u***||*_p_* = ⟨***u***,***u***⟩*_p_*^1/2^. If the curvature of M is constant at all points, such as on a sphere or hyperboloid, the matrix form of *g_p_* is constant.

The ordered pair (M, *g_p_*) defines a *Riemannian manifold*. In the following sections, only Riemannian manifolds will be considered due to their metric properties.

A *geodesic* γ : [0,1] 🡪 M is a generalization of a Euclidean straight line to Riemannian manifolds. The shortest path between points *p* and *q* on M is along a geodesic connecting the points. For example, on the sphere or the surface of the earth, geodesics are great circles passing through a pair of points. Distances between points along geodesics are denoted dist_M_(·,·).

Tangent space vectors are transported along geodesics using the *Parallel Transport* function τ*_p_*_🡪_*_q_*: *T_p_*M 🡪 *T_q_*M (Figure S1, center). Parallel transport is a norm-preserving map linking the tangent spaces of M. More precisely, the Riemannian metric between vectors ***v***, ***u*** from *T_p_*M is preserved, such that ⟨***v***, ***u***⟩*_p_* = ⟨τ*_p_*_🡪_*_q_*(***v***), τ*_p_*_🡪_*_q_*(***u***)⟩*_q_*.

The *Exponential Map* function exp*_p_* : *T_p_*M 🡪 M maps a small perturbation around *p* to a point on M along a geodesic determined by the direction of a tangent vector (Figure S1, right). Let γ : [0,1] 🡪 M be a geodesic such that γ(0) = *p*, γ(1) = *q*, and γ’(0) = ***u*** is a vector from *T_p_*M. The Exponential Map is then defined as exp*_p_*(***u***) = γ(1). Notably, the distance along γ in M is preserved by exp*_p_*, such that ||***u***||*_p_* = dist_M_(*p*, *q*).

The exact of form of the Exponential Map and Parallel Transport functions depend on the manifold and will be given below for hyperbolic manifolds.

**S3. Hyperbolic Geometry**

Hyperbolic geometry is the study of spaces with constant negative curvature. This is in contrast with Euclidean or spherical geometries with zero or positive curvature, respectively. Intuitively, the branching structure of a tree can be thought of as a discrete realization of a hyperbolic space. In this section and the following, this connection will be developed within a rigorous framework to enable precise mappings from the local coordinates output by hPCA to a latent negatively-curved manifold.

Hyperbolic spaces cannot be isometrically embedded within Euclidean space, leading to fundamental difficulties in understanding and visualizing negatively-curved space. Consequently, many models of hyperbolic geometry have been developed. These include the Poincaré disk 𝔻, upper half-plane 𝕌, and hyperboloid ℍ, among others. Each model emphasizes different aspects of hyperbolic geometry, but no model completely encapsulates all of its features. For instance, the hyperboloid model facilitates calculations within hyperbolic spaces, but its visualization is challenging. In contrast, the Poincaré disk and upper half-plane models allow for convenient visualization, but more complicated and numerically unstable calculations. Fortunately, all of these models are equivalent in the sense that there are isometric transformations between them. This allows each model to be used in its suitable domain and subsequently mapped to a different domain as needed. In the following, we develop an efficient method for projecting points and vectors within hyperbolic spaces, based on the hyperboloid model of hyperbolic geometry. This method will be used to map the output of hPCA onto the manifold ℍ*^n^*.

To construct the hyperboloid model of ℍ*^n^* embedded within an ambient space, we define the Lorentzian inner product ⟨·,·⟩*_H_* and associated norm ||·||*_H_* by

|  | $\left\langle\boldsymbol{x}, \boldsymbol{y} \right\rangle_{H}=-x_{0}y_{0}+\sum_{i=1}^{n} x_{i}y_{i}, \boldsymbol{x}, \boldsymbol{y}\in\mathbb{R}^{n+1},$  $\left\Vert\boldsymbol{x} \right\Vert_{H}=\sqrt{\left\langle\boldsymbol{x}, \boldsymbol{x} \right\rangle_{H}}, \left\Vert\boldsymbol{x} \right\Vert_{H}\mathbb{\in C,}$ | (S1) |
| --- | --- | --- |

where vector indices are counted starting from zero. The space ℝ*^n^*^+1^ equipped with the above inner product is a (*n*+1)-dimensional Minkowski space. Note that, unlike the Euclidean inner product, the Lorentzian inner product can be negative.

The *n*-dimensional hyperboloid ℍ*^n^* is the collection of all points such that,

|  | $\mathbb{H}^{n}=\left\{ \boldsymbol{x}\in\mathbb{R}^{n+1} \vert\left\langle\boldsymbol{x}\mathbf{,}\boldsymbol{x} \right\rangle_{H}=-1, x_{0}>0 \right\}.$ | (S2) |
| --- | --- | --- |

For *n* = 1, equation (S2) describes a standard hyperbola resulting from conic sections. That is, ℍ^1^ is the intersection of a cone with a plane in ℝ^3^, with the branches of the hyperbola curving around the *x*_0_ axis. In higher dimensions, the equation describes a negatively-curved hypersurface centered at the point *o* = [1,0,…,0], located near the origin of ℝ*^n^*^+1^.

Distances between points *x* and *y* on ℍ*^n^* with coordinates ***x***, ***y*** within the ambient Minkowski space, are calculated using hyperbolic trigonometry,

|  | $\mathrm{dist}_{H}\left( x\mathbf{,}y \right)=\cosh^{-1} \left( \left\langle\boldsymbol{x}\mathbf{,}\boldsymbol{y} \right\rangle_{H} \right) , x,y\in\mathbb{H}^{n}.$ | (S3) |
| --- | --- | --- |

The shortest path between any pair of points on ℍ*^n^* is along a geodesic, with a total length calculated by equation (S3).

**S4. Operations on Hyperbolic Manifolds**

The tools of differential geometry outlined in section S2 can be applied to hyperbolic spaces. Since ℍ*^n^* is a locally-smooth embedding within ℝ*^n^*^+1^ at all points, it is a manifold. The Riemannian metric on ℍ*^n^* is the diagonal matrix *g_H_* = diag(-1,1,…,1). Consequently, (ℍ*^n^*, *g_H_*) is a Riemannian manifold and the tools of differential geometry can be applied.

The tangent space of ℍ*^n^* at point *p* with coordinate representation ***p*** is defined as,

|  | $T_{p}\mathbb{H}^{n}=\left\{ \boldsymbol{u}\in\mathbb{R}^{n+1} \vert\left\langle\boldsymbol{u}\mathbf{,}\boldsymbol{p} \right\rangle_{H}=0 \right\}.$ | (S4) |
| --- | --- | --- |

Notably, vectors in *T_p_*ℍ*^n^* are not orthogonal to ***p*** in the Euclidean sense (e.g., ⟨***u***,***p***⟩ = 0), but instead follow the closely-related definition in equation (S4). Accordingly, the tangent space *T_p_*ℍ*^n^* can be described as all vectors from the ambient space ℝ*^n^*^+1^ that are hyperbolically-orthogonal to *p*. Each tangent space inherits the Lorentzian inner product (S1) from the ambient space. Furthermore, this inner product is positive-definite when restricted to vectors from a tangent space.

Vectors within a subspace of ℝ*^n^* can be easily embedded into *T_o_*ℍ*^n^* , the tangent space at the central point of the hyperbola *o* = [1,0,…,0]. Let ***u’*** be a subspace vector, formatted as a row vector to simplify notation. It is embedded within ℝ*^n^*^+1^ using the inclusion function *ι_o_*: ℝ*^k^* 🡪 *T_o_*ℍ*^n^* defined by,

|  | $\iota_{o}\left( \boldsymbol{u}^{\boldsymbol{'}} \right)=\left[ 0,\boldsymbol{u}^{\boldsymbol{'}}\mathbf{,0} \right], \boldsymbol{u}^{\boldsymbol{'}}\in\mathbb{R}^{k},$ | (S5) |
| --- | --- | --- |

where **0** is a vector of *n* – *k* zeros. Note that this embedding preserves lengths within the ambient space. Specifically, if ***u*** = *ι_o_*(***u’***), then ||***u’***|| = ||***u***|| = ||***u***||*_H_*.

The Parallel Transfer function τ*_p_*_🡪_*_q_*: *T_p_*ℍ*^n^* 🡪 *T_q_*ℍ*^n^* is given by,

|  | $\tau_{p\to q}\left( \boldsymbol{u} \right)=\boldsymbol{u}+\frac{\left\langle\boldsymbol{q}\mathbf{,}\boldsymbol{u} \right\rangle_{H}}{1\mathbf{-}\left\langle\boldsymbol{p}\mathbf{,}\boldsymbol{q} \right\rangle_{H}}\left( \boldsymbol{p}+\boldsymbol{q} \right)\boldsymbol{, u}\boldsymbol{\in}T_{p}\mathbb{H}^{n}.$ | (S6) |
| --- | --- | --- |

Since Parallel Transfer preserves lengths, ||***u***||*_H_* = ||τ*_p_*_🡪_*_q_* (***u***)||*_H_*. Additionally, equation (S6) gives a relatively simple method for calculating the coordinates of vectors in the tangent space of arbitrary points on ℍ*^n^*. Specifically, the vector is first embedded into the tangent space *T_o_*ℍ*^n^* using (S5), then parallel transported to the tangent space of any point on the manifold using (S6).

Lastly, the Exponential Map used to map *T_p_*ℍ*^n^* to a point on ℍ*^n^* is given by

|  | $\exp_{p}\left( \boldsymbol{u} \right)=\cosh\left( \left\Vert\boldsymbol{u} \right\Vert_{H} \right) \boldsymbol{p}+\sinh\left( \left\Vert\boldsymbol{u} \right\Vert_{H} \right)\frac{\boldsymbol{u}}{\left\Vert\boldsymbol{u} \right\Vert_{H}}, \boldsymbol{u}\boldsymbol{\in}T_{p}\mathbb{H}^{n}\mathbf{.}$ | (S7) |
| --- | --- | --- |

Since the Exponential Map preserves lengths, ||***u***||*_H_* = dist*_H_*(exp*_p_*(***u***), ***p***).

The above equations, in combination, facilitate calculations on ℍ*^n^*. A vector from a locally Euclidean coordinate system is embedded into the tangent space at the center *o* using (S5). Subsequently, the embedded vector is then transported to the tangent space of an arbitrary point using (S6), preserving its hyperbolic length. Finally, the vector is projected onto the manifold using (S7), again preserving its hyperbolic length. The length of the original vector is preserved at every step in this process. Furthermore, since the Riemannian metric is unchanged, all steps locally preserve angles. Consequently, angles between variables and data points in the same tangent space are preserved by the embedding and projection into ℍ*^n^*.

In the main text, the above functions were used to construct the composite function *ϕ_i,p_*: SO(*k*) x ℝ*^k^* 🡪 ℍ*^k^*,

|  | $\varphi_{i,p}=\exp_{p}\circ\tau_{o\to p}\circ\iota_{o}\circ\Delta_{i},$ | (S8) |
| --- | --- | --- |

where Δ*_i_*: SO(*k*) x ℝ*^k^* 🡪 ℝ*^k^* is the displacement function displacement coordinate function detailed in Appendix A of the main text. This composite function was used in Algorithm 2 to embed the matrix of eigenvectors from SO(*k*) and vector of eigenvalues from ℝ*^k^* from a local PCA onto the hyperboloid ℍ*^k^* and subsequently projected onto the Poincaré disk 𝔻.

**References:**

1. Lee, J. M. (2006). *Riemannian manifolds: an introduction to curvature*. Springer.

2. Lee, J. M. (2012). *Introduction to Smooth Manifolds*. Springer.
