# Supplementary figures and images for: Rootlets Hierarchical Principal Component Analysis for Revealing Nested Dependencies in Hierarchical Data
Source: Mathematics (Basel). Author manuscript; Available in PMC 2025 Sep 24. (PMC12456745; doi:10.3390/math13010072)

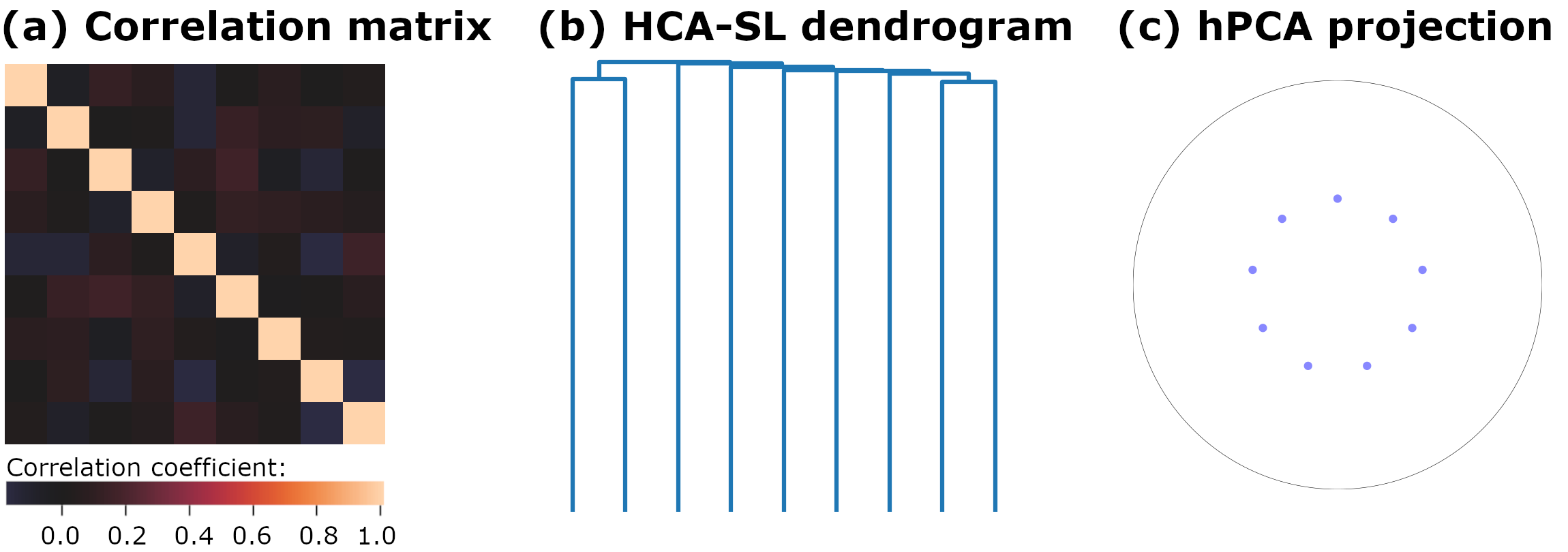

Supplement: Figure S2: Single linkage Hierarchical Clustering Analysis for Experiment 1 [file NIHMS2060439-supplement-Figure_S2__Single_linkage_Hierarchical_Clustering_Analysis_for_Experiment_1.tiff]

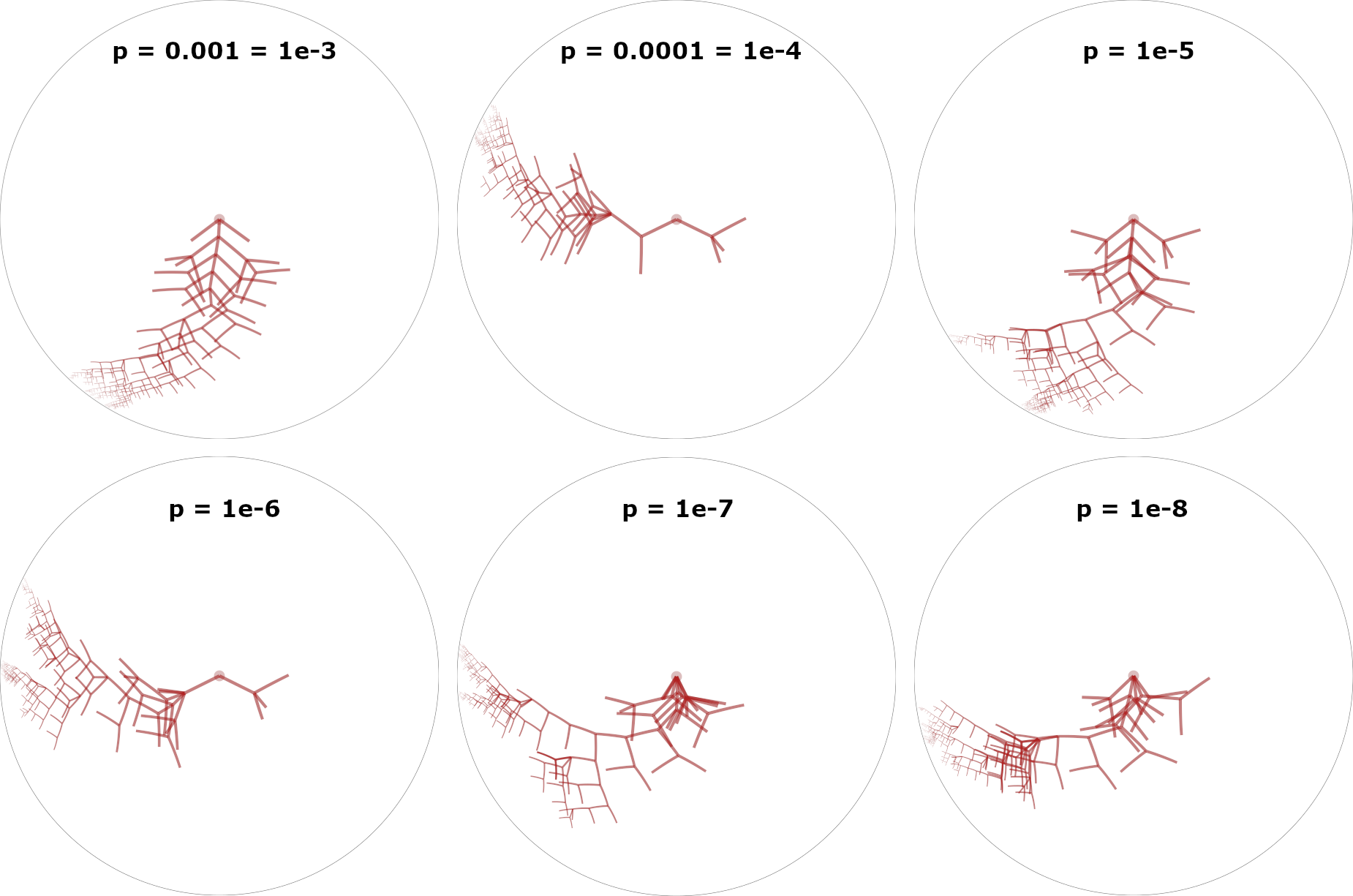

Supplement: Figure S3: Rootlets hPCA p-value cutoff parameter comparison [file NIHMS2060439-supplement-Figure_S3__Rootlets_hPCA_p-value_cutoff_parameter_comparison.tiff]
